# Responses of Free-Living *Vibrio* Community to Seasonal Environmental Variation in a Subtropical Inland Bay

**DOI:** 10.3389/fmicb.2020.610974

**Published:** 2020-12-14

**Authors:** Xing Chen, Huaxian Zhao, Gonglingxia Jiang, Jinli Tang, Qiangsheng Xu, Lengjinghua Huang, Si Chen, Shuqi Zou, Ke Dong, Nan Li

**Affiliations:** ^1^Key Laboratory of Environment Change and Resources Use in Beibu Gulf, Ministry of Education, Nanning Normal University, Nanning, China; ^2^State Key Laboratory for Conservation and Utilization of Subtropical Agro-Bioresources, Guangxi Microorganism and Enzyme Research Center of Engineering Technology, College of Life Science and Technology, Guangxi University, Nanning, China; ^3^Department of Biological Sciences, Kyonggi University, Suwon-si, South Korea

**Keywords:** 16S rRNA, *Vibrio* diversity, seasonal variation, eutrophication, Beibu Gulf

## Abstract

*Vibrio* are widely distributed in aquatic environments and strongly associated with eutrophic environments and human health through the consumption of contaminated seafood. However, the response of the *Vibrio* community to seasonal variation in eutrophic environments is poorly understood. In this study, we used a *Vibrio*-specific 16S rRNA sequencing approach to reveal the seasonal distribution pattern and diversity of the *Vibrio* community in the Maowei Sea, Beibu Gulf of China. The Shannon diversity of the *Vibrio* community was highest in the summer, while β-diversity analysis showed that *Vibrio* community structures were significantly different between seasons. Distance-based redundancy analysis (dbRDA) and Mantel test analysis suggested that total dissolved nitrogen (TDN), total dissolved phosphorus (TDP), dissolved inorganic nitrogen (DIN), salinity, and temperature were the key environmental factors shaping the *Vibrio* community structure, indicating a strong filtering effect of trophic condition on *Vibrio* communities. Furthermore, through random forest analysis, *V. fluvialis*, *V. alginolyticus*, *V. proteolyticus*, *V. splendidus*, and the other eight *Vibrio* species were more sensitive to eutrophic changes. This study revealed seasonal changes in *Vibrio* communities and the influence of environmental variation on *Vibrio* community composition, contributing to a better understanding of their potential ecological roles in a subtropical inland bay.

## Introduction

The genus *Vibrio* consists of heterotrophic bacteria that are common in marine ecosystems, especially in coastal areas (Westrich, 2015; Shelford and Suttle, 2018; Zhang et al., 2019). Due to their remarkable capability for decomposition of a range of nutrient resources, including chitin and alginate, *Vibrio* may exert large impacts on biogeochemical cycling in coastal habitats (Kopprio et al., 2017; Zhang et al., 2018). Furthermore, the genus *Vibrio* encompasses many facultatively symbiotic and potential pathogenic strains, such as *Vibrio cholerae*, *Vibrio parahaemolyticus*, and *Vibrio vulnificus*, which are all capable of infecting humans through the consumption of contaminated seafood (Guin et al., 2019; hun Yoon and Waters, 2019; King et al., 2019). Since they drive major biogeochemical cycles and support food webs globally, *Vibrio* communities are a vital component of the marine ecosystem. Furthermore, the diversity and dynamics of the *Vibrio* community have attracted growing interest worldwide, including in the Northern Chinese marginal seas, coastal North Atlantic, and Gulf Coast of Mexico (López-Hernández et al., 2015; Vezzulli et al., 2016; Wang et al., 2019). Understanding when and where pathogenic *Vibrio* will break out is important for aquaculture and marine health. A range of studies (Hartwick et al., 2019; Lamon et al., 2019; Baddam et al., 2020) have indicated that the *Vibrio* community undergoes substantial temporal variation. However, the response of the *Vibrio* community to seasonal variation in eutrophic environments is still poorly understood.

*Vibrio* communities are highly diverse and complex and vary between habitats due to changing environmental conditions. This increases the necessity of studying the ecology of *Vibrio*. Previous studies have demonstrated that the dynamics of *Vibrio* populations are linked to multiple factors, including seasonal changes, biological parameters, and physicochemical properties such as temperature, salinity, pH, and dissolved oxygen (DO), as well as associations with nutrients (Urquhart et al., 2016; Greenfield et al., 2017; Froelich et al., 2019). For example, Siboni et al. (2016) revealed that shifts in the composition of the *Vibrio* community between seasons were primarily driven by changes in temperature, salinity, and NO_2_^–^. Takemura et al. (2014) found temperature-associated increases in the abundance of pathogenic *Vibrio* species observed in Australian coastal areas. Considering that the occurrence of specific vibrios has frequently been linked to the temperature, salinity, and nutrients, we hypothesized that seasonal variability in the coastal ecosystem could characterize the level of *Vibrio* community differentiation under ongoing environmental forcing.

Moreover, *Vibrio* disease events and community structure appear to be inextricably linked to anthropogenic disturbance of the aquatic environment. An influx of anthropogenically derived pollutants and nutrients into aquatic systems significantly disrupts the *Vibrio* community structure, contributing to changes in diversity and an increase in potentially pathogenic *Vibrio* species (Shore-Maggio et al., 2018). For example, the dense human population and low-lying geography of an estuarine environment were hypothesized to have allowed the enrichment of *V. cholerae*, which causes fatal damage to the human gut (Boucher et al., 2015). Besides, *Vibrio* are strongly associated with high nutrient environments, which are considered a potential indicator for environmental changes and eutrophication (Gregoracci et al., 2012; Takemura et al., 2014). Consequently, gaining deeper insight into the diversity of marine *Vibrio* and the role of environmental factors as filters of *Vibrio* community composition will help people evaluate their ecological role and public health effect in the marine coastal ecosystem.

To better understand the associations between the free-living *Vibrio* community and eutrophic perturbation, we collected surface water samples from the Maowei Sea, which is an estuarine-bay ecosystem that suffers from anthropogenic activities and receives large inputs of terrestrial nutrients from the QinJiang and Maoling rivers, existing serious eutrophication in the area (Zhu et al., 2019). Therefore, this research presented here focuses on the associations between *Vibrio* community composition and seasonal patterns under broadly eutrophic condition. Our aims were to (a) fill the gap in knowledge of the seasonal composition and variation in the *Vibrio* community in the Maowei Sea, (b) determine the gradients of the key controlling environmental factors driving the dynamics of the *Vibrio* community, (c) and identify the potential indicative *Vibrio* species for eutrophic conditions in the local ecosystem.

## Materials and Methods

### Sampling Sites and Environmental Parameters

**FIGURE 1:**
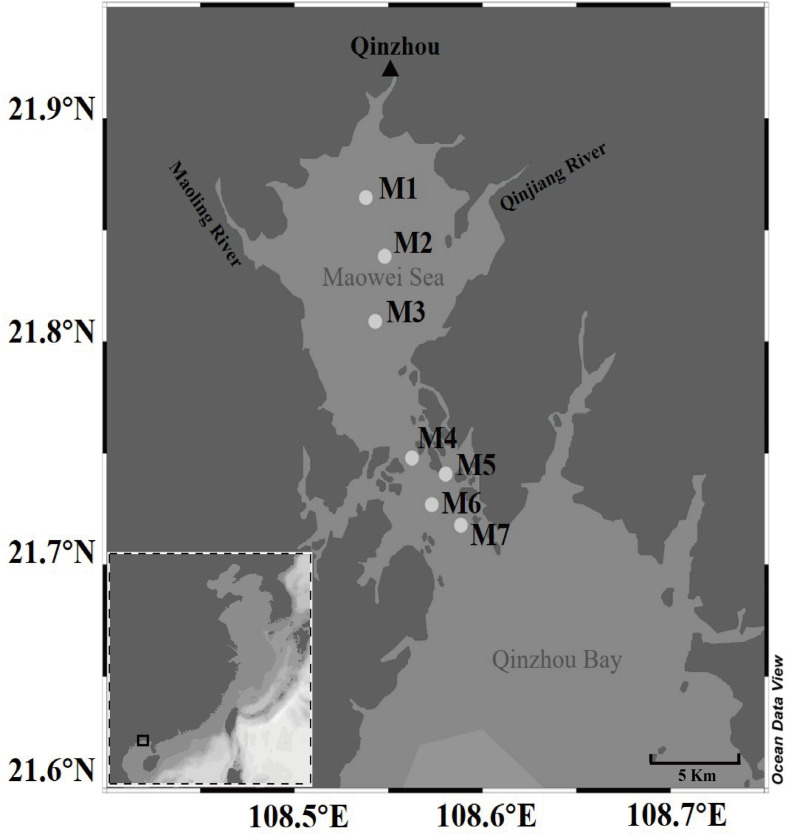
Map of the seven sampling sites along the Maowei Sea in the Beibu Gulf of China.

We collected a total of 140 surface seawater samples (0.5 m deep) seasonally from seven sites of the Maowei Sea from June 2017 through March 2018 (Figure 1). Two liters of seawater was collected from four corners and the middle of a 5 m × 5 m square area in each site by a five-point sampling method. Thereafter, all seawater samples were stored at 4°C on board and transferred to the laboratory before filtration. A vacuum pump was used to sequentially filter 1 L of seawater per sample through 3-μm filters (Port Washington, NY, United States) to remove debris and larger organisms, and the resulting samples were collected on 0.22-μm-pore-size polycarbonate membranes (Millipore Corporation, Billerica, MA, United States) for subsequent analysis. During sampling, seawater temperature, pH, and salinity in each sample were measured using a portable meter (556 MPS; YSI, United States). Other nutrients in each sample were measured by standard methods previously described by Yao et al. (2014). For example, dissolved inorganic nutrients (NO_2_^–^, NO_3_^–^, and NH_4_^+^) were measured using spectrophotometric and colorimetric methods (Han et al., 2012). Dissolved inorganic nitrogen (DIN) was composed of NO_2_^–^, NO_3_^–^, and NH_4_^+^, and dissolved inorganic phosphorus (DIP) represented the concentration of PO_4_-P (Lai et al., 2014). Chl *a* was measured using a fluorescence spectrophotometer (Holm-Hansen and Riemann, 1978). The total organic carbon (TOC) and the chemical oxygen demand (COD) were measured using the standard method (Visco et al., 2005). The total dissolved nitrogen (TDN) and the total dissolved phosphorus (TDP) were determined using the Cu–Cd column reduction and the spectrophotometric phosphomolybdate blue method, respectively (Sah, 1994; Cai and Guo, 2009). The eutrophication index (EI) was indicated as a Chinese eutrophication status index, which was calculated as the following formula: EI = DIN × DIP × COD × 10^6^/4500 (Lai et al., 2014; Chen et al., 2016). According to the degree of eutrophication, we found that the samples could be classified into three eutrophication statues: low eutrophication (1 < EI < 3), medium eutrophication (3 < EI < 9), and high eutrophication (EI > 9) (Supplementary Table 1). Samples nearby the ocean area showed the lower eutrophication, whereas samples located next to the river mouth presented higher eutrophication.

### DNA Extraction and PCR Amplification

Genomic DNA extraction was performed by a DNeasy PowerWater Kit (QIAGEN, United States) using 0.22-μm-pore-size polycarbonate membranes according to the manufacturer’s protocols. DNA yield and purity were evaluated using a Nanodrop-2000 Spectrophotometer (Thermo Scientific, United States). The DNA sample was preserved at −80°C. The extracted DNA was tested by PCR using the *Vibrio*-specific 16S rRNA primers 169F (5′-GGCGTAAAGCGCATGCAGGT-3′) and 680R (5′-GAAATTCTACCCCCCTCTACAG-3′) (Thompson et al., 2004). A total reaction volume of 20 μL PCR mixture containing 2 μL DNA template, 6 μL ddH_2_O, 10 μL 2 × Taq PCR Mastermix (TIANGEN, China), and 2 μL forward and reverse primers were conducted on a Bio-Rad thermocycler (Hercules, CA). The amplification process was as follows: an initial activation step at 94°C for 1 min, followed by 35 cycles of 30 s denaturation at 95°C, annealing at 56°C for 30 s, extension at 72°C for 30 s, and a final elongation step at 72°C for 10 min. Ultrapure water was used instead of a sample solution as a negative control, which ruled out the possibility of false-positive PCR results. The PCR products were verified by 2% agarose gel electrophoresis and visualized with a UV light and gel image system.

### High-Throughput Sequencing Processing

Sequencing was performed on the Illumina MiSeq platform of Lianchuan Bio-Technology Company (China) after preparing DNA libraries. Sequences with low-quality reads (quality scores <30) were removed using Trimmomatics (Bolger et al., 2014). Adapter and barcode sequences were eliminated by Cutadapt (Martin, 2011). The paired-end demultiplexed sequencing reads were processed in a QIIME2 environment (Bolyen et al., 2019). Using a VSEARCH plugin (Rognes et al., 2016), the paired-end reads were joined followed by dereplicating, chimeras filtering, and operational taxonomic units (OTUs) clustering (*de novo*) at a 97% sequence similarity level. Taxonomic classification of OTUs was assigned by a local Blastn (cutoff E-value 1e-10) against a Ribosomal Database Project (RDP) database (Release 11) (Cole et al., 2014).

### Statistical Analysis

Seasonal differences in environmental parameters were evaluated using a one-way analysis of variance (ANOVA) followed by Tukey’s HSD test in SPSS Statistics v24.0 (Subtype and Sloan, 2018). The α-diversity indices between different groups of samples, including Shannon, Simpson, Chao1, and observed number of OTUs were calculated using the vegan package in R (Dixon, 2003). The β-diversity was performed with Bray-Curtis dissimilarity, principal coordinate analysis (PCoA), ANOSIM, and PERMANOVA for comparison between the seasonal *Vibrio* communities. Random forest analysis was used to examine the important indicator taxa. Spearman correlation, Mantel test analysis, redundancy analysis (RDA), variation partitioning analysis (VPA), and linear regression were also run in R to reveal correlations between the *Vibrio* community and environmental factors. The R software packages used in this study are mainly the “vegan,” “random forest,” and “psych” packages (R Core Team, 2013). Analysis of the quantitative and evolutionary relationships of *Vibrio* species in different seasons was performed using TBtool (Chen C. et al., 2018).

## Results

### Variation in Environmental Parameters

The environmental parameters of seawater samples collected from the Maowei Sea in the Beibu Gulf were measured during the seasonal cruises (Supplementary Table 1). The environmental parameters of the seawater samples all varied significantly between seasons as per ANOVA (Tukey’s HSD test, *p* < 0.001) (Table 1). The temperature (24.58 ± 6.25°C), Chl *a* (2.23 ± 1.10 μg/L), TOC (1.20 ± 0.29 mg/L), and COD (2.82 ± 0.69 mg/L) were significantly higher in summer and autumn than in spring and winter. In contrast, the value for salinity (18.19 ± 5.13), DO (7.64 ± 1.37 mg/L), and TDP (0.06 ± 0.01 mg/L) showed higher in spring and winter. In TDN, there were differences between fall and winter, with the lowest value (0.57 ± 0.07) in winter and the highest value (0.79 ± 0.180) in fall. Moreover, NO_2_^–^, NO_3_^–^, and NH_4_^+^ concentrations all showed lower values in winter. According to EI indicators, the eutrophication level was significantly lowest in winter and highest in fall. Also, within each season, the eutrophication showed a decreasing spatial trend from the river mouth site M1 to the open sea site M7 (Supplementary Table 1). Overall, environmental parameters varied between all the sites, and the eutrophication conditions showed significant seasonal changes (*p* < 0.001; Table 1).

**TABLE 1:** One-way ANOVA test on variation of each water chemical parameter in year-round.

Variables	*P*-values	All		Spring		Summer		Fall		Winter	
		Mean	Std. Dev.	Mean	Std. Dev.	Mean	Std. Dev.	Mean	Std. Dev.	Mean	Std. Dev.
Temp	<0.001	24.588	6.245	24.119	0.692	27.820	0.842	31.524	0.854	14.890	0.471
pH	<0.001	7.629	0.285	7.784	0.214	7.776	0.249	7.474	0.258	7.483	0.252
Salinity	<0.001	18.193	5.130	21.222	1.862	18.398	4.231	12.281	5.443	20.871	1.932
DO	<0.001	7.641	1.367	8.992	0.318	6.430	0.480	6.232	0.393	8.910	0.266
NO_2_^–^	<0.001	0.024	0.025	0.015	0.005	0.010	0.004	0.062	0.022	0.006	0.002
NO_3_^–^	<0.001	0.340	0.157	0.376	0.045	0.519	0.123	0.314	0.102	0.149	0.036
NH_4_^+^	<0.001	0.111	0.047	0.143	0.027	0.115	0.034	0.122	0.059	0.064	0.009
Chl *a*	<0.001	2.227	1.106	1.607	0.417	2.809	1.299	3.100	0.861	1.390	0.385
TDN	<0.001	0.690	0.144	0.765	0.060	0.636	0.105	0.790	0.180	0.569	0.067
DIN	<0.001	0.474	0.181	0.535	0.074	0.644	0.102	0.498	0.125	0.219	0.044
DIP	<0.001	0.029	0.014	0.044	0.011	0.015	0.004	0.038	0.006	0.018	0.005
TDP	<0.001	0.061	0.019	0.084	0.004	0.039	0.010	0.058	0.011	0.064	0.011
e	<0.001	1.197	0.285	0.938	0.149	1.317	0.183	1.470	0.277	1.065	0.144
COD	<0.001	2.818	0.691	2.508	0.424	3.330	0.554	3.218	0.566	2.255	0.513
EI	<0.001	9.385	6.877	13.632	6.203	7.125	2.108	14.543	6.650	2.242	1.577

*Temp, temperature; DO, dissolved oxygen; Chl *a*, chlorophyll A; TDN, total dissolved nitrogen; DIN, dissolved inorganic nitrogen; DIP, dissolved inorganic phosphorus; TDP, total dissolved phosphorus; TOC, total organic carbon; COD, chemical oxygen demand; EI, eutrophication index.*

### Correlation of *α*-Diversity With Environmental Factors

**FIGURE 2:**
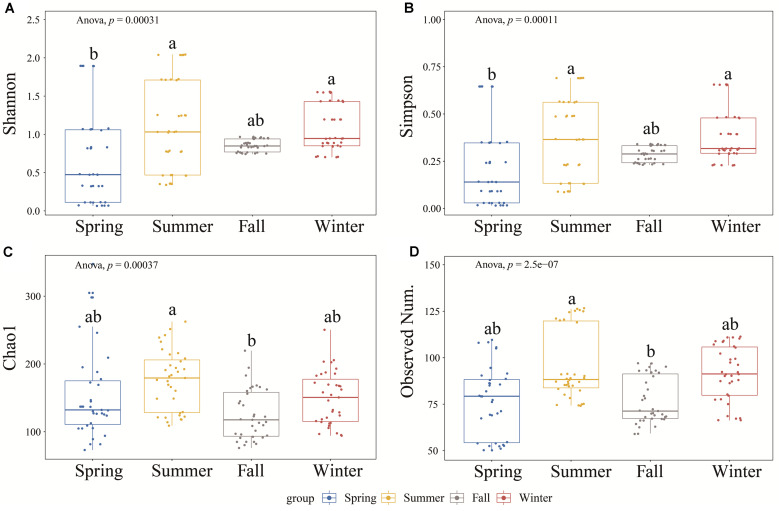
The α-diversity indices (**A–D**: Shannon, Simpson, Chao 1, and Observed Number of OTUs) among the four seasons by boxplot. The differences between any two groups were tested by Tukey’s HSD test, presented by lowercase letters on the plots.

A total of 12,589 ± 5,735 sequences were generated from the samples after quality control and clustered into 85 ± 18 OTUs at a 97% similarity level (Supplementary Table 2). Good’s coverage values were greater than 99%, indicating that the current sequences reflected the actual situation of the majority of the *Vibrio* community. The α-diversity of the *Vibrio* community including Shannon, Simpson, Chao1, and the observed number of OTU indices varied and differed significantly across seasons (ANOVA, *p* < 0.001) (Figure 2). The summer group showed the highest α-diversity, including Shannon, Simpson, and Chao1 indices, indicating that evenness and richness were significantly higher than in other seasons (Figure 2). However, the Shannon and Simpson diversities of the *Vibrio* community were lowest in the spring samples and significantly different from summer and winter. According to Spearman’s correlation analysis, the α-diversity of the *Vibrio* community was negatively (*p* < 0.01) correlated to NO_2_^–^, NO_3_^–^, NH_4_^+^, TDN, DIN, DIP, and COD but only positively correlated to salinity (*p* < 0.001) (Supplementary Table 3). Since the EI is a synthetic index of the multiple nutrients of DIN (NO_2_^–^, NO_3_^–^, and NH_4_^+^), DIP, and COD, we determined that the occurrence of eutrophication, especially in summer and fall, was accompanied by a decrease in the α-diversity of the *Vibrio* community. In addition, TDN, DIN, NO_2_^–^, and salinity were the most influential (| r| > 0.28, *p* < 0.001) among these factors.

### Seasonal Changes in *Vibrio* Community Composition

**FIGURE 3:**
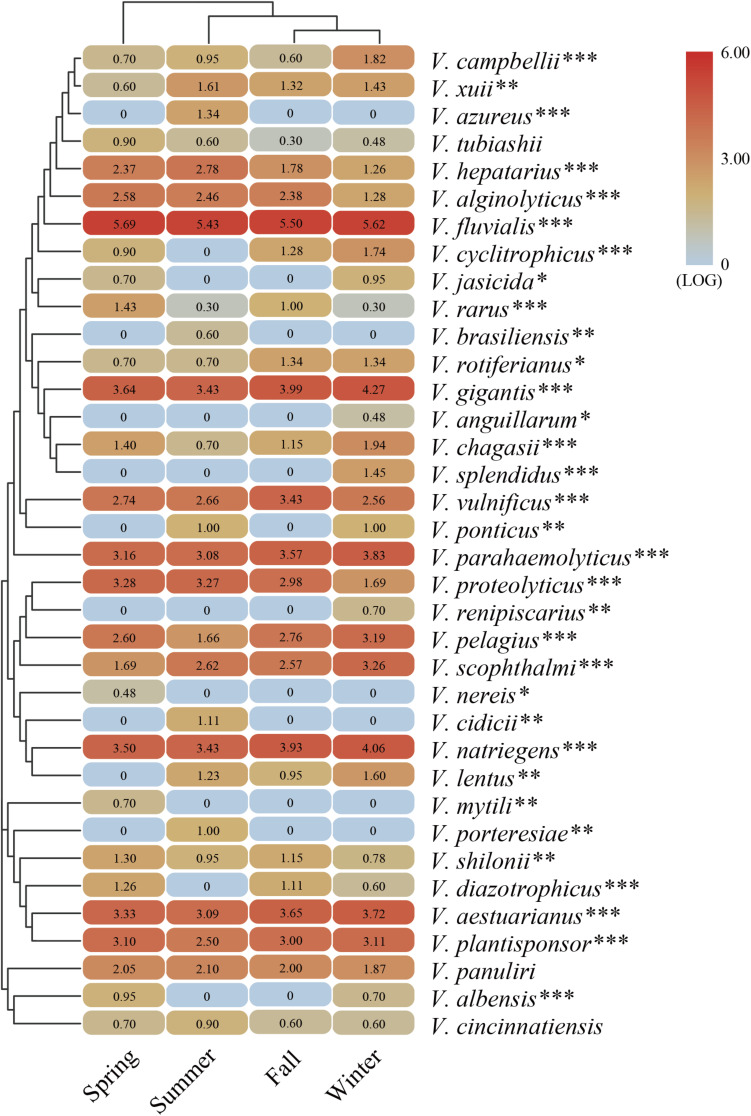
The heat map generated from the major abundant *Vibrio* species with representative sequences in four seasons. The color code represents the difference in relative abundance, ranging from blue to red. The top tree shows the cluster relationship of sampling groups. The significant differences between seasonal changes and distribution of *Vibrio* species are presented by one-way ANOVA test (*p < 0.05; **p < 0.01; ***p < 0.001).

The representative sequences of each OTU were analyzed to determine the taxonomic status and calculate the relative abundance (Supplementary Figure 1). The results showed that the total sequences were affiliated with 36 known *Vibrio* species. *V. fluvialis*, *V. gigantis*, *V. natriegens*, *V. aestuarianus*, *V. plantisponsor*, *V. proteolyticus*, and *V. parahaemolyticus* were predominant in all seasonal samples. *V. fluvialis* was the most abundant species occupying a large proportion (>50%) of each sample. The variation in community composition was largely attributable to the presence of *V. fluvialis* that dominated the *Vibrio* community. Meanwhile, a considerable proportion (∼10%) of the sequence was assigned to an unclassified group, which might represent novel *Vibrio* species. To better understand the seasonal differences of the *Vibrio* species, a heat map was generated to reflect the relative abundance among the four seasonal groups (Figure 3). A total of 21 *Vibrio* species were detected in all four seasonal groups, whereas 15 *Vibrio* species were found to be seasonally dependent. For example, *V. azureus*, *V. brasiliensis*, *V. cidicii*, and *V. porteresiae* existed only in summer, while *V. anguillarum*, *V. renipiscarius*, and *V. splendidus* were only found in winter and *Vibrio nereis and V. mytili* were only found in spring. In addition, *V. cyclitrophicus* and *V. diazotrophicus* existed widely in all groups except the summer group. The cluster cladogram analysis in the heat map illustrated that the fall and winter samples grouped together, while groups of spring and summer were clustered separately. Moreover, the main abundant *Vibrio* species were significantly correlated with the seasonal changes via ANOVA (Figure 3). The above results suggested that the *Vibrio* community composition was significantly different seasonally and followed a seasonal distribution pattern in the Beibu Gulf.

### Effects of Environmental Variables on the *β*-Diversity

**FIGURE 4:**
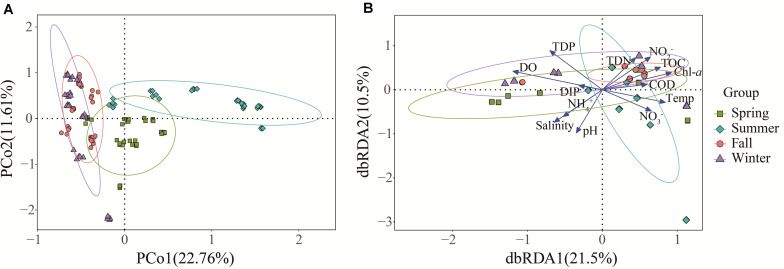
The β-diversity of the *Vibrio* community and its relationship with environmental factors in four seasons. **(A)** The PCoA of different samples based on Bray-Curtis dissimilarity of *Vibrio* communities. **(B)** The dbRDA plot shows the relationship between samples and environmental factors.

**FIGURE 5:**
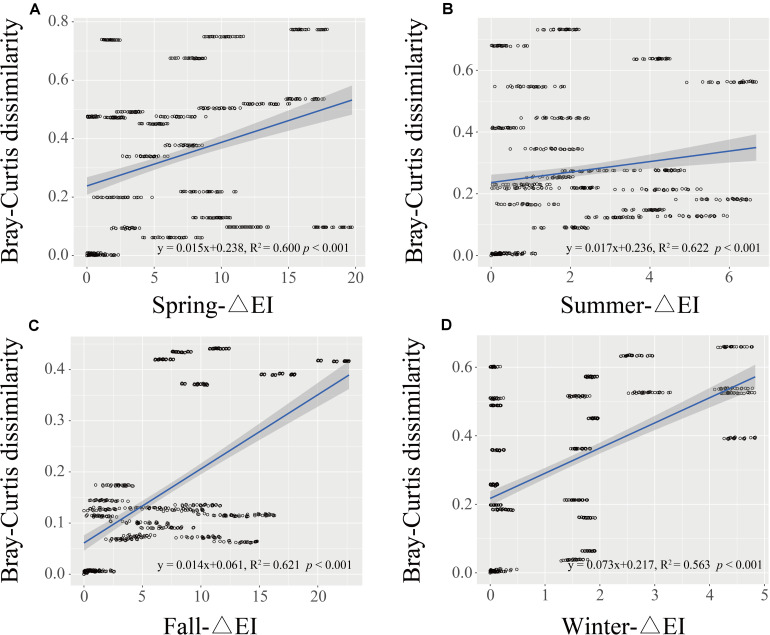
The regression between Bray-Curtis dissimilarity and variation of Delta EI in four seasons (**A–D** represents spring to winter). The black line represents the linear regression, and *P*-values were calculated to indicate significant differences.

Principal coordinate analysis (PCoA) based on the OTU level was performed to investigate the β-diversity of the *Vibrio* community in different seasonal groups (Figure 4A). The first two PCoA axes explained 22.76 and 11.61% of the total variation, respectively. The results demonstrated that summer samples were clearly separated from the others, indicating that the *Vibrio* community composition differed strongly between the summer and the other groups, whereas the *Vibrio* community of fall and winter groups was similar, which was consistent with the cluster cladogram (Figure 3). Both ANOSIM (*r* = 0.33, *p* = 0.001) and PERMANOVA (*r*^2^ = 0.29, *p* = 0.001) tests further confirmed that the differences in *Vibrio* community structures between the four seasons were significant. Furthermore, the Bray-Curtis dissimilarity showed that the dissimilarity of the *Vibrio* community structure among the different samples was highest in spring and lowest in fall (Supplementary Figure 2). The significant differences in the Bray-Curtis dissimilarity between seasons also further reflected the seasonal difference of the *Vibrio* community structure in the Maowei sea. To further determine the relationship between the *Vibrio* community dissimilarity and the eutrophic changes, linear regressions were performed based on Bray-Curtis dissimilarity and showed significantly increasing β-diversity correlated with the eutrophic variations in seasons (Figure 5).

**FIGURE 6:**
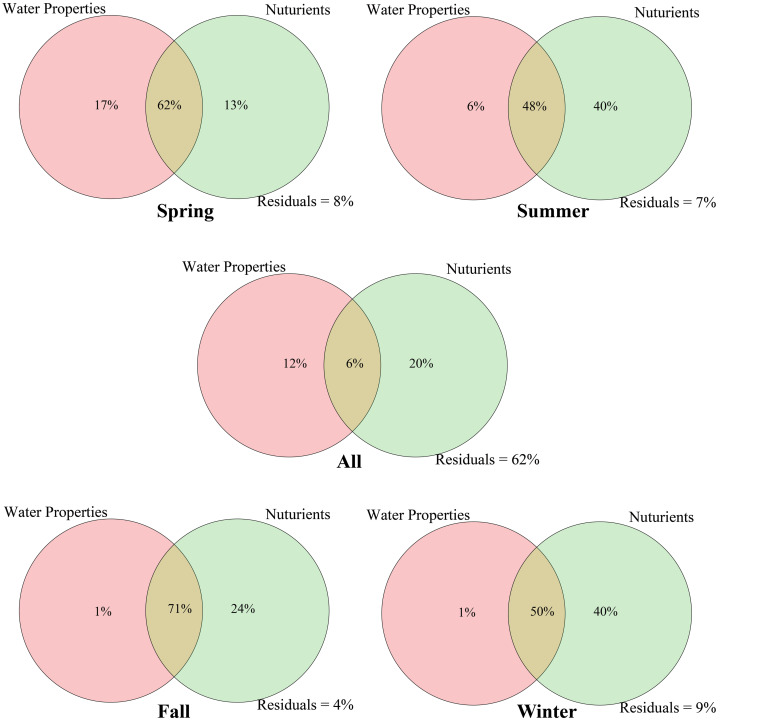
Variation partitioning analysis shows the effects of water properties (temperature, pH, salinity, DO, Chl *a*, and COD) and nutrients (NO_2_^–^, NO_3_^–^, NH_4_^+^, TDN, TDP, TOC, DIN, and DIP) on the *Vibrio* community.

Variation partitioning analysis (VPA) was used to analyze the effects of nutrient variables (NO_2_^–^, NO_3_^–^, NH_4_^+^, TDN, TDP, TOC, DIN, and DIP) and water properties (temperature, pH, salinity, DO, Chl *a*, and COD) on seasonal *Vibrio* community heterogeneity. The results showed that the pure effect of nutrient variables (20%) was higher than that of water properties (12%), and the combined effects explained 38% of the total community variation (Figure 6). In seasons, the pure effects of nutrient variables were almost all higher than those of water properties, except spring. For example, in winter samples, the pure effect of nutrient variables was 40%, and the combined effects explained of the total community variation was 91%. The dbRDA was performed to determine the specific environmental factors that governed the *Vibrio* community structure in different seasons. The dbRDA suggested that almost all environmental factors were strongly correlated with *Vibrio* community structures (Figure 4B), and shifts in the *Vibrio* community structure may be due to main environmental factors, such as TDN, TDP, DO, temperature, and salinity (Supplementary Table 4). Furthermore, the Mantel test calculated the correlation between environmental factors and the β-diversity of the *Vibrio* community (Table 2). The partial Mantel results showed that the *Vibrio* community composition along environmental gradients was significantly correlated with TDN, TDP, temperature, and DO, with shifts in community composition being better correlated with changes in TDP than TDN.

**TABLE 2:** The mantel and partial mantel tests (Pearson) show the correlations between Bray-Curtis dissimilarity of the *Vibrio* community and the environmental factors (*p < 0.05; **p < 0.01; ***p < 0.001).

Environmental factors	Whole		Spring		Summer		Fall		Winter	
	Mantel	Partial mantel	Mantel	Partial mantel	Mantel	Partial mantel	Mantel	Partial mantel	Mantel	Partial mantel
Temp	0.120***	0.114***	–0.061	–0.120	0.003	–0.021	–0.066	–0.064	0.051	0.056
pH	0.064*	0.055	–0.009	–0.023	0.258**	0.081	0.001	0.014	–0.016	0.010
Salinity	–0.024	–0.076	0.294**	0.357***	0.763***	0.686***	0.493***	0.441***	0.181**	–0.064
DO	0.225***	0.207***	0.594***	0.564***	0.264**	0.126	–0.077	–0.105	0.092	0.085
NO_2_^–^	–0.104	–0.156	0.330***	0.294***	0.673***	0.566***	0.456***	0.376***	0.485***	0.271***
NO_3_^–^	0.074*	0.049	0.661***	0.753***	0.861***	0.789***	0.150*	0.050	0.537***	0.402***
NH_4_^+^	0.009	–0.043	0.006	–0.378	0.021	–0.404	0.284***	0.124*	0.179**	–0.035
Chl *a*	–0.011	–0.049	0.090	–0.289	0.135*	–0.202	0.578***	0.562***	0.280***	0.206**
TDN	0.124**	0.085*	0.780***	0.783***	0.700***	0.383***	–0.061	–0.079	0.407***	0.296***
DIN	0.110***	0.073*	0.764***	0.786***	0.657***	0.178**	0.219**	0.075	0.482***	0.322***
DIP	0.103**	0.073*	0.615***	0.591***	0.906***	0.853***	0.024	–0.250	0.394***	0.062
TDP	0.200***	0.200***	0.334***	0.278***	–0.039	–0.086	0.135*	0.068	0.419***	0.334***
TOC	0.077*	0.036	0.493***	0.481***	0.794***	0.607***	0.509***	0.444***	0.269***	–0.075
COD	0.032	–0.014	0.570***	0.625***	0.210**	–0.606	0.470***	0.398***	0.324***	–0.012
EI	0.045	0.005	0.067	–0.182	0.528***	–0.288	0.244**	–0.022	0.472***	0.426***

### Indicator Species of *Vibrio* for Eutrophication

**FIGURE 7:**
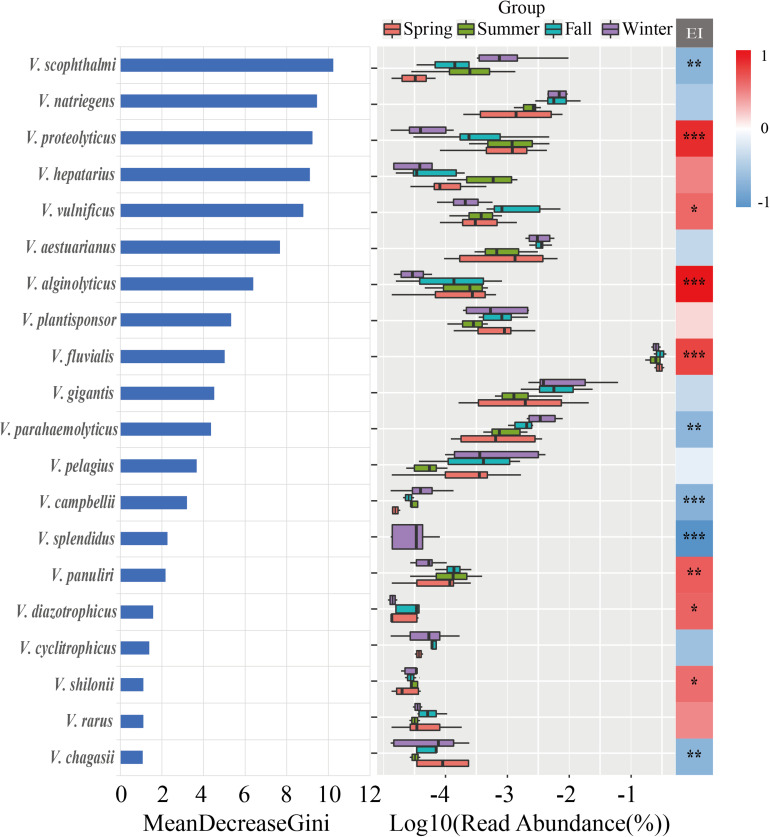
The random forest analysis of the top 20 important *Vibrio* species, ranked by Gini index. Middle: ranked by the richness of the top 20 *Vibrio* species with boxplot. Right: the correlations between the abundances of top 20 *Vibrio* species and EI variable by Spearman correlation analysis. *p < 0.05; **p < 0.01; ***p < 0.001.

The random forest method was utilized to analyze the most important *Vibrio* species for classifying the seasonal samples, indicating that *V. scophthalmi*, *V. natriegens*, *V. proteolyticus*, *V. hepatarius*, and *V. vulnificus* were the most important species with relatively high abundances (Figure 7). Furthermore, linear regression analysis was used to explore the trend of sensitive *Vibrio* species with EI (Supplementary Figure 3). The results revealed that 12 of the top 20 important *Vibrio* species were significantly correlated with the EI values. For example, *V. splendidus* was negatively correlated with EI changes (*R*^2^ = 0.535, *p* < 0.001), while *V. fluvialis* (*R*^2^ = 0.662, *p* < 0.001), *V. alginolyticus* (*R*^2^ = 0.576, *p* < 0.001), and *V. proteolyticus* (*R*^2^ = 0.386, *p* < 0.001) were positively correlated with the degree of eutrophication. These *Vibrio* species from the region with trophic gradients may be more sensitive to the eutrophication, which can assess whether nutrient contents had posed an important selective constraint on the communities.

## Discussion

This study investigated the seasonal variation in *Vibrio* diversity in a subtropical coastal sea system. Analysis of *Vibrio*-specific 16S rRNA sequences showed that *V. fluvialis* was present year-round and occupied the largest proportion of the *Vibrio* community, followed by *V. gigantis*, *V. natriegens*, and *V. parahaemolyticus* (Figure 3). *V. fluvialis* is known as an emerging foodborne pathogen, which was isolated from marine and estuarine environments and humans exhibiting severe diarrheal disease indicating its considerable adaptability (Ramamurthy et al., 2014). In Toulon harbor, France, detections of *V. fluvialis* have increased considerably (29.3%) in seawater during the process of filter feeding in marine mollusks (Martin and Bonnefont, 1990). Similarly, *V. fluvialis* was predominantly detected (>50%) in all samples, indicating the higher possibility and risk of causing gastroenteritis in humans year-round in the studied area. The second most abundant group was *V. gigantis*, which has been reported to originate from the hemolymph of diseased cultured oysters (Le Roux et al., 2005). The Maowei sea is a natural ecosystem rich in wild oyster populations, providing an opportunity for the bloom of pathogenic *Vibrio* species such as *V. fluvialis*, *V. gigantis*, *V. parahaemolyticus*, and *V. vulnificus*, which infect humans through oyster consumption (Chen X. et al., 2018). These may also lead to frequent outbreak of diseases in aquatic organisms and thereby impair aquaculture economy. Moreover, the relative abundance of the different *Vibrio* species varied between the different seasons, and several *Vibrio* species detected in this study showed season-specific prevalence, including *V. brasiliensis* and *V. splendidus*, which were detected only in summer and winter, respectively (Figure 3). These results indicated that *Vibrio* communities from the four seasons exhibited contrasting community compositions and were sensitive to seasonal environmental changes.

Regarding α-diversity, we elucidated the temporal changes in *Vibrio* diversity in the Beibu Gulf. The α-diversity of *Vibrio* was highest in summer, which is consistent with previous research in which the diversity of *Vibrio* was found to be greater in summer than in winter (Siboni et al., 2016). Meanwhile, the results of β-diversity revealed distinct seasonal changes in the *Vibrio* community composition of the Maowei Sea and the summer group showing a significant difference from the others on the PCoA (Figure 4A). From spring to summer, Shannon and Simpson diversities increased significantly, and the *Vibrio* community structure in summer was significantly different from those in other seasons. This could be partly due to the arrival of the wet season and an increase in runoff. Conversely, it could be that summer is the fastest growing season for shellfish, increasing their filter-feeding rate, and a large number of metabolites were excreted as the water temperature rose (Zheng et al., 2012). Moreover, one possible explanation for the seasonal fluctuation pattern of Bray-Curtis dissimilarity (high in spring and winter and low in summer and autumn) is that the river inflows and seawater temperature increase in summer can stimulate the rapid growth of the motile, chemotactic species of *Vibrio* making them the dominant species (Figure 5). This may be the cause of the higher similarity of the *Vibrio* community in summer and fall. When exposed to cold seawater in winter, *Vibrio* enter into a viable but non-culturable state, leading to the persistence of various *Vibrio* species (Oliver, 2016). In addition, previous studies have suggested that marine bacterial diversity increased with the input of human-derived pollution and nutrients, such as N, P, and C, which could contribute to the creation of favorable conditions for the proliferation of different microorganisms (Nogales et al., 2011; Numberger et al., 2019). Therefore, our results were consistent with these studies, indicating that nutrient variations may lead to the increase in *Vibrio* diversity in the Beibu Gulf.

We observed that the changes in the *Vibrio* community composition and diversity in relation to seasons were more likely to be a result of a direct influence of temperature, salinity, TDN, DIN, and TDP. Our findings confirmed that salinity was the most important environmental determinant of α-diversity in the *Vibrio* community as described in previous studies (Siboni et al., 2016; Wang et al., 2019), and observed a similar positive correlation (Spearman, *p* < 0.001). The Bray-Curtis dissimilarity of the *Vibrio* community was also significantly correlated with temperature in the Maowei sea (Table 2 Mantel test, *p* < 0.001) providing further evidence that warmer seawater increased the dissimilarity of the *Vibrio* community (Liang et al., 2019). Except for the temperature and salinity, RDA and Mantel test analysis indicated that the roles of TDN and TDP were significantly positively correlated with the seasonal variation in the *Vibrio* community (Figure 4 and Table 2). Moreover, the regression analysis showed that *Vibrio* community dissimilarity was significantly increased with the variation in main environmental factors (TDN, TDP, salinity, and DO) (Supplementary Figure 4). The positive correlation between DO and *Vibrio* community dissimilarity demonstrated that the relative accumulation of dissolved oxygen in seawater enhanced the heterogeneity of the *Vibrio* community, which was consistent with previous studies (Liang et al., 2019; Wang et al., 2019). With these environmental factors becoming the main factors constraining the community composition, the pool of *Vibrio* species that would have been capable of surviving these high trophic conditions and able to outcompete and replace less adapted ones may have decreased. In this study, the VPA result also revealed that the effects of nutrients were stronger than those of water properties, indicating that the variations of nitrogen and phosphorus availably shaped and filtered the observed variations in the *Vibrio* community.

High concentrations of TDN, TDP, and DIN were closely related to the occurrence of eutrophication (Ko et al., 2019). Recently, the eutrophication in the Maowei sea could be attributed to land-based runoff and pollution produced by aquaculture in the area due to a lack of water exchange and large quantities of oyster excreta (Yang et al., 2019). Particularly in summer, the discharge from the Qinjiang River and Maoling River into the Maowei sea led to a high concentration of nutrients (Lai et al., 2014). Consequently, the input of the nutrients led to the *Vibrio* community showing the highest diversity in summer and significant seasonal variation. Besides, previous studies found that the *Vibrio* diversity was increased with nutritional levels, suggesting that *Vibrio* species could be considered as indicators of trophic conditions (Gregoracci et al., 2012; Mansergh and Zehr, 2014). In this study, we identified 12 *Vibrio* species such as *V. fluvialis*, *V. proteolyticus*, *V. alginolyticus*, and *V. splendidus* that had significant positive or negative relationships with the eutrophication (Figure 7 and Supplementary Figure 3). For example, *V. fluvialis* has the capacity to utilize a wide range of organic and inorganic nutrients containing C, N, and P (such as L-arginine, L-arabinose, D-glucose, chitin, malonate, phosphate, and citrate) (Brenner et al., 1983). This strong capacity for nutrient utilization may cause increased population levels in these species with increased pollution levels. By contrast, *V. splendidus* is a pathogen associated with oysters (Vezzulli et al., 2015). The degradation and destruction of the host due to overfishing and eutrophication in the nearshore region may cause the loss of associated microbiomes. In summary, discovery of these indicative species may facilitate the assessment of a trophic shift and its subsequent ecological effects.

## Conclusion

This study demonstrated that highly diverse *Vibrio* communities were present in the Maowei Sea with great temporal resolution. Additionally, our study observed that the variation in *Vibrio* diversity and community composition was mainly affected by changes of salinity, temperature, nitrogen, and phosphorus (TDN, TDP, and DIN). Several important *Vibrio* species, such as *V. splendidus*, *V. proteolyticus*, *V. fluvialis*, and *V. alginolyticus* were significantly correlated with trophic changes, which may be more sensitive to eutrophic status in coastal ecosystems. This investigation enhanced our understanding of the *Vibrio* seasonal distribution in response to environmental factors under eutrophication pressure and their ecological effects and public health in a subtropical inland bay.

## Data Availability Statement

The datasets presented in this study can be found in online repositories. The names of the repository/repositories and accession number(s) can be found below: https://www.ncbi.nlm.nih.gov/, PRJNA607420.

## Author Contributions

NL and HZ conceived and designed the experiments. XC performed the experiments and analyzed the data. XC, GJ, JT, QX, LH, SC, SZ, and KD wrote and helped increase the quality of this manuscript. All authors contributed to the article and approved the submitted version.

## Conflict of Interest

The authors declare that the research was conducted in the absence of any commercial or financial relationships that could be construed as a potential conflict of interest.
