# Supplementary material for: Responses of Free-Living Vibrio Community to Seasonal Environmental Variation in a Subtropical Inland Bay
Source: Front Microbiol. 2020 Dec 14;11:610974. doi: 10.3389/fmicb.2020.610974 (PMC7767907; doi:10.3389/fmicb.2020.610974)
Supplement: Supplementary file 1 [file Data_Sheet_1.docx]

**Supplementary materials**

**Responses of free-living *Vibrio* community to seasonal environmental variation in** **a subtropical inland bay**

**Xing Chen^1,2^, Huaxian Zhao^1^, Gonglingxia Jiang^1^, Jinli Tang^1^, Qiangsheng Xu^1^, Lengjinghua Huang^1^, Si Chen^2^, Shuqi Zou^3^, Ke Dong^3^, Nan Li^1*^**

^1^ Key Laboratory of Environment Change and Resources Use in Beibu Gulf, Ministry of Education (Nanning Normal University), 175 Mingxiu East Road, Nanning, Guangxi, 530001, China

^2^ State Key Laboratory for Conservation and Utilization of Subtropical Agro-bioresources, Guangxi Microorganism and Enzyme Research Center of Engineering Technology, College of Life Science and Technology, Guangxi University, 100 Daxue East Road, Nanning, Guangxi, 530004, China

^3^ Department of Biological Sciences, Kyonggi University, 154-42, Gwanggyosan-ro, Yeongtong-gu, Suwon-si, Gyeonggi-do, 16227, South Korea

*** Correspondence:**Nan Li, nli0417@163.com

**Table S1 Sampling sites and environmental parameters**

| **Season** | **Sampling Date** | **Sample Codes** | **Temp (℃)** | **pH** | **Salinity (ppt)** | **DO** | **NO_2_^-^** | **NO_3_^-^** | **NH_4_^+^** | **Chl *a*** | **TDN** | **DIN** | **DIP** | **TDP** | **TOC** | **COD** | **EI** | **EI level** |
| --- | --- | --- | --- | --- | --- | --- | --- | --- | --- | --- | --- | --- | --- | --- | --- | --- | --- | --- |
|  |  |  |  |  |  | mg/L | mg/L | mg/L | mg/L | µg/L | mg/L | mg/L | mg/L | mg/L | mg/L | mg/L | mg/L |  |
| **Summer** | **June 10,2017** | SU1.1 | 27.338 | 7.285 | 11.627 | 6.557 | 0.016 | 0.692 | 0.066 | 5.253 | 0.762 | 0.773 | 0.013 | 0.051 | 1.578 | 4.289 | 9.230 | **High** |
|  |  | SU1.2 | 29.545 | 7.752 | 11.868 | 6.293 | 0.016 | 0.701 | 0.063 | 5.341 | 0.772 | 0.779 | 0.013 | 0.053 | 1.604 | 4.036 | 9.058 |  |
|  |  | SU1.3 | 27.366 | 7.476 | 11.374 | 6.590 | 0.014 | 0.707 | 0.063 | 5.682 | 0.768 | 0.784 | 0.013 | 0.053 | 1.639 | 3.957 | 9.008 |  |
|  |  | SU1.4 | 29.602 | 7.729 | 10.960 | 6.570 | 0.015 | 0.695 | 0.063 | 5.319 | 0.802 | 0.773 | 0.012 | 0.055 | 1.628 | 4.358 | 9.271 |  |
|  |  | SU1.5 | 29.149 | 7.675 | 11.029 | 6.656 | 0.016 | 0.664 | 0.066 | 5.500 | 0.765 | 0.745 | 0.013 | 0.052 | 1.606 | 4.247 | 9.081 |  |
|  |  | SU2.1 | 27.292 | 7.407 | 13.398 | 6.665 | 0.015 | 0.607 | 0.066 | 3.966 | 0.674 | 0.687 | 0.019 | 0.025 | 1.461 | 3.806 | 11.115 |  |
|  |  | SU2.2 | 27.662 | 7.939 | 13.678 | 7.057 | 0.015 | 0.579 | 0.067 | 3.725 | 0.687 | 0.661 | 0.019 | 0.027 | 1.397 | 3.707 | 10.335 |  |
|  |  | SU2.3 | 29.451 | 8.016 | 14.266 | 6.787 | 0.015 | 0.616 | 0.069 | 3.935 | 0.694 | 0.699 | 0.020 | 0.025 | 1.362 | 3.714 | 11.352 |  |
|  |  | SU2.4 | 27.008 | 7.923 | 14.322 | 6.598 | 0.015 | 0.599 | 0.072 | 3.892 | 0.710 | 0.687 | 0.019 | 0.026 | 1.481 | 3.847 | 11.133 |  |
|  |  | SU2.5 | 28.627 | 7.400 | 14.588 | 6.625 | 0.015 | 0.625 | 0.070 | 3.892 | 0.713 | 0.710 | 0.019 | 0.025 | 1.344 | 3.839 | 11.447 |  |
|  |  | SU3.1 | 27.876 | 7.424 | 17.172 | 6.965 | 0.011 | 0.573 | 0.109 | 2.719 | 0.612 | 0.693 | 0.012 | 0.035 | 1.395 | 3.206 | 6.164 | **Medium** |
|  |  | SU3.2 | 27.076 | 8.141 | 17.298 | 7.193 | 0.011 | 0.577 | 0.107 | 2.568 | 0.623 | 0.695 | 0.013 | 0.035 | 1.320 | 3.374 | 6.541 |  |
|  |  | SU3.3 | 28.069 | 7.891 | 17.604 | 6.778 | 0.011 | 0.587 | 0.109 | 2.616 | 0.598 | 0.707 | 0.014 | 0.032 | 1.366 | 3.377 | 7.215 |  |
|  |  | SU3.4 | 28.345 | 7.681 | 18.216 | 6.985 | 0.011 | 0.604 | 0.103 | 2.765 | 0.621 | 0.718 | 0.013 | 0.035 | 1.330 | 3.401 | 7.004 |  |
|  |  | SU3.5 | 27.766 | 7.704 | 17.604 | 6.833 | 0.011 | 0.568 | 0.103 | 2.743 | 0.584 | 0.682 | 0.013 | 0.036 | 1.383 | 3.508 | 6.777 |  |
|  |  | SU4.1 | 28.578 | 7.798 | 18.259 | 6.920 | 0.009 | 0.529 | 0.139 | 2.040 | 0.616 | 0.677 | 0.012 | 0.040 | 1.362 | 3.343 | 5.941 |  |
|  |  | SU4.2 | 27.049 | 7.510 | 19.532 | 6.847 | 0.009 | 0.555 | 0.142 | 2.036 | 0.599 | 0.706 | 0.013 | 0.038 | 1.378 | 3.353 | 6.613 |  |
|  |  | SU4.3 | 26.883 | 8.148 | 19.494 | 6.413 | 0.009 | 0.527 | 0.139 | 1.940 | 0.593 | 0.675 | 0.012 | 0.040 | 1.385 | 3.260 | 5.824 |  |
|  |  | SU4.4 | 28.134 | 7.829 | 18.715 | 6.627 | 0.009 | 0.530 | 0.133 | 2.010 | 0.601 | 0.671 | 0.011 | 0.040 | 1.282 | 3.221 | 5.500 |  |
|  |  | SU4.5 | 27.939 | 7.494 | 19.931 | 6.707 | 0.009 | 0.516 | 0.136 | 1.912 | 0.640 | 0.661 | 0.012 | 0.041 | 1.364 | 3.436 | 6.144 |  |
|  |  | SU5.1 | 28.161 | 7.861 | 21.893 | 5.910 | 0.006 | 0.445 | 0.161 | 1.946 | 0.633 | 0.612 | 0.013 | 0.043 | 1.159 | 2.826 | 5.015 |  |
|  |  | SU5.2 | 27.105 | 7.861 | 22.703 | 6.202 | 0.006 | 0.432 | 0.160 | 1.928 | 0.624 | 0.598 | 0.014 | 0.043 | 1.110 | 2.876 | 5.162 |  |
|  |  | SU5.3 | 27.689 | 8.086 | 22.028 | 5.910 | 0.006 | 0.441 | 0.161 | 2.080 | 0.685 | 0.609 | 0.013 | 0.042 | 1.145 | 2.803 | 4.921 |  |
|  |  | SU5.4 | 26.799 | 7.465 | 21.398 | 5.964 | 0.006 | 0.437 | 0.155 | 1.990 | 0.637 | 0.598 | 0.013 | 0.040 | 1.147 | 2.809 | 5.040 |  |
|  |  | SU5.5 | 27.605 | 7.690 | 22.860 | 6.086 | 0.006 | 0.442 | 0.161 | 2.076 | 0.671 | 0.610 | 0.014 | 0.040 | 1.204 | 3.079 | 5.682 |  |
|  |  | SU6.1 | 26.275 | 7.523 | 20.275 | 6.186 | 0.009 | 0.493 | 0.131 | 2.108 | 0.668 | 0.633 | 0.014 | 0.048 | 1.302 | 3.427 | 6.746 |  |
|  |  | SU6.2 | 26.855 | 8.169 | 20.193 | 6.760 | 0.009 | 0.485 | 0.132 | 2.231 | 0.662 | 0.626 | 0.014 | 0.050 | 1.368 | 3.536 | 6.980 |  |
|  |  | SU6.3 | 27.655 | 7.920 | 20.111 | 6.566 | 0.009 | 0.476 | 0.135 | 2.277 | 0.719 | 0.620 | 0.013 | 0.048 | 1.305 | 3.550 | 6.579 |  |
|  |  | SU6.4 | 27.738 | 7.593 | 20.111 | 6.173 | 0.009 | 0.514 | 0.127 | 2.127 | 0.717 | 0.650 | 0.014 | 0.050 | 1.342 | 3.536 | 6.910 |  |
|  |  | SU6.5 | 28.676 | 7.982 | 21.054 | 6.760 | 0.009 | 0.499 | 0.126 | 2.288 | 0.689 | 0.635 | 0.014 | 0.050 | 1.282 | 3.469 | 6.716 |  |
|  |  | SU7.1 | 28.179 | 8.014 | 25.032 | 5.601 | 0.005 | 0.282 | 0.132 | 1.487 | 0.439 | 0.419 | 0.023 | 0.028 | 1.024 | 2.291 | 4.860 |  |
|  |  | SU7.2 | 28.458 | 8.122 | 23.544 | 5.640 | 0.005 | 0.290 | 0.138 | 1.482 | 0.444 | 0.433 | 0.022 | 0.029 | 0.956 | 2.327 | 5.007 |  |
|  |  | SU7.3 | 26.924 | 7.866 | 24.096 | 5.274 | 0.005 | 0.291 | 0.134 | 1.463 | 0.410 | 0.431 | 0.021 | 0.027 | 1.026 | 2.351 | 4.821 |  |
|  |  | SU7.4 | 28.095 | 8.068 | 22.848 | 5.629 | 0.005 | 0.292 | 0.145 | 1.514 | 0.411 | 0.442 | 0.023 | 0.029 | 1.023 | 2.423 | 5.409 |  |
|  |  | SU7.5 | 26.728 | 7.719 | 24.840 | 5.739 | 0.005 | 0.287 | 0.132 | 1.463 | 0.406 | 0.424 | 0.022 | 0.028 | 1.024 | 2.310 | 4.770 |  |
| **Fall** | **September 9,2017** | FA1.1 | 31.916 | 7.242 | 6.017 | 6.592 | 0.055 | 0.416 | 0.225 | 4.563 | 1.030 | 0.695 | 0.041 | 0.065 | 1.825 | 4.127 | 26.247 | **High** |
|  |  | FA1.2 | 31.758 | 7.534 | 6.111 | 6.225 | 0.052 | 0.401 | 0.242 | 4.611 | 1.042 | 0.695 | 0.042 | 0.064 | 1.764 | 4.050 | 26.269 |  |
|  |  | FA1.3 | 31.379 | 7.548 | 6.161 | 6.434 | 0.055 | 0.395 | 0.231 | 4.616 | 1.109 | 0.682 | 0.041 | 0.063 | 1.807 | 3.867 | 23.981 |  |
|  |  | FA1.4 | 32.959 | 6.942 | 6.180 | 6.269 | 0.052 | 0.407 | 0.234 | 4.250 | 1.125 | 0.693 | 0.041 | 0.065 | 1.752 | 4.005 | 25.330 |  |
|  |  | FA1.5 | 30.557 | 7.132 | 6.590 | 6.371 | 0.053 | 0.398 | 0.242 | 4.312 | 1.094 | 0.693 | 0.044 | 0.063 | 1.724 | 3.908 | 26.357 |  |
|  |  | FA2.1 | 30.210 | 7.051 | 6.700 | 6.363 | 0.041 | 0.408 | 0.157 | 3.739 | 0.836 | 0.606 | 0.039 | 0.040 | 1.716 | 3.866 | 20.083 |  |
|  |  | FA2.2 | 32.651 | 7.721 | 7.176 | 6.357 | 0.041 | 0.398 | 0.160 | 3.865 | 0.817 | 0.599 | 0.037 | 0.041 | 1.608 | 3.874 | 19.138 |  |
|  |  | FA2.3 | 32.714 | 7.463 | 6.555 | 6.279 | 0.043 | 0.434 | 0.158 | 3.743 | 0.827 | 0.635 | 0.038 | 0.039 | 1.582 | 4.075 | 21.722 |  |
|  |  | FA2.4 | 30.115 | 7.404 | 7.155 | 6.286 | 0.044 | 0.420 | 0.152 | 3.732 | 0.863 | 0.616 | 0.040 | 0.042 | 1.662 | 3.764 | 20.692 |  |
|  |  | FA2.5 | 32.588 | 7.154 | 6.997 | 6.376 | 0.044 | 0.427 | 0.150 | 3.621 | 0.827 | 0.621 | 0.041 | 0.039 | 1.657 | 3.878 | 21.677 |  |
|  |  | FA3.1 | 30.720 | 7.289 | 8.872 | 6.894 | 0.092 | 0.368 | 0.060 | 2.808 | 0.907 | 0.520 | 0.037 | 0.054 | 1.652 | 3.563 | 15.113 |  |
|  |  | FA3.2 | 30.977 | 7.480 | 8.947 | 6.653 | 0.087 | 0.366 | 0.055 | 2.808 | 0.885 | 0.508 | 0.038 | 0.052 | 1.594 | 3.473 | 14.728 |  |
|  |  | FA3.3 | 31.940 | 7.716 | 9.728 | 6.854 | 0.093 | 0.366 | 0.061 | 2.800 | 0.882 | 0.520 | 0.038 | 0.054 | 1.609 | 3.700 | 16.107 |  |
|  |  | FA3.4 | 32.293 | 7.289 | 8.993 | 6.747 | 0.093 | 0.370 | 0.061 | 2.892 | 0.885 | 0.523 | 0.036 | 0.051 | 1.629 | 3.599 | 15.217 |  |
|  |  | FA3.5 | 32.228 | 7.441 | 9.337 | 6.914 | 0.087 | 0.364 | 0.056 | 2.789 | 0.897 | 0.507 | 0.040 | 0.050 | 1.679 | 3.509 | 15.625 |  |
|  |  | FA4.1 | 31.934 | 7.175 | 11.200 | 6.260 | 0.069 | 0.346 | 0.066 | 2.310 | 0.779 | 0.480 | 0.041 | 0.051 | 1.582 | 3.119 | 13.681 |  |
|  |  | FA4.2 | 31.965 | 7.258 | 11.200 | 6.227 | 0.067 | 0.336 | 0.067 | 2.099 | 0.752 | 0.469 | 0.043 | 0.056 | 1.627 | 2.966 | 13.409 |  |
|  |  | FA4.3 | 31.840 | 7.512 | 11.267 | 6.590 | 0.067 | 0.346 | 0.065 | 2.134 | 0.755 | 0.478 | 0.044 | 0.053 | 1.579 | 3.074 | 14.253 |  |
|  |  | FA4.4 | 31.023 | 7.190 | 10.856 | 6.240 | 0.066 | 0.333 | 0.063 | 2.273 | 0.729 | 0.462 | 0.042 | 0.052 | 1.629 | 2.890 | 12.339 |  |
|  |  | FA4.5 | 30.521 | 7.662 | 10.911 | 6.804 | 0.065 | 0.340 | 0.069 | 2.130 | 0.801 | 0.474 | 0.043 | 0.052 | 1.600 | 3.153 | 14.162 |  |
|  |  | FA5.1 | 31.326 | 7.527 | 15.562 | 5.620 | 0.076 | 0.263 | 0.106 | 2.408 | 0.733 | 0.446 | 0.041 | 0.058 | 1.468 | 2.819 | 11.541 |  |
|  |  | FA5.2 | 32.825 | 7.288 | 15.314 | 6.090 | 0.074 | 0.259 | 0.104 | 2.375 | 0.710 | 0.437 | 0.044 | 0.059 | 1.390 | 2.730 | 11.609 |  |
|  |  | FA5.3 | 30.784 | 7.228 | 15.531 | 5.626 | 0.070 | 0.266 | 0.102 | 2.535 | 0.707 | 0.439 | 0.042 | 0.059 | 1.487 | 2.929 | 12.021 |  |
|  |  | FA5.4 | 32.570 | 7.617 | 15.206 | 5.551 | 0.070 | 0.274 | 0.103 | 2.493 | 0.727 | 0.446 | 0.044 | 0.060 | 1.510 | 2.946 | 12.852 |  |
|  |  | FA5.5 | 32.315 | 7.617 | 15.206 | 5.655 | 0.075 | 0.274 | 0.100 | 2.498 | 0.709 | 0.448 | 0.045 | 0.059 | 1.432 | 2.768 | 12.342 |  |
|  |  | FA6.1 | 30.486 | 7.750 | 13.914 | 6.246 | 0.083 | 0.305 | 0.086 | 2.288 | 0.828 | 0.474 | 0.040 | 0.081 | 1.238 | 2.571 | 10.713 |  |
|  |  | FA6.2 | 31.488 | 7.337 | 14.743 | 6.167 | 0.085 | 0.309 | 0.083 | 2.281 | 0.783 | 0.477 | 0.038 | 0.081 | 1.298 | 2.811 | 11.343 |  |
|  |  | FA6.3 | 30.424 | 7.390 | 13.914 | 6.118 | 0.085 | 0.306 | 0.086 | 2.090 | 0.829 | 0.477 | 0.039 | 0.078 | 1.224 | 2.593 | 10.729 |  |
|  |  | FA6.4 | 31.519 | 7.855 | 14.829 | 6.228 | 0.084 | 0.302 | 0.086 | 2.121 | 0.774 | 0.472 | 0.038 | 0.078 | 1.289 | 2.630 | 10.412 |  |
|  |  | FA6.5 | 31.613 | 7.743 | 14.843 | 6.295 | 0.087 | 0.310 | 0.087 | 2.167 | 0.761 | 0.484 | 0.036 | 0.080 | 1.232 | 2.590 | 10.061 |  |
|  |  | FA7.1 | 32.225 | 7.753 | 21.990 | 5.807 | 0.024 | 0.102 | 0.152 | 3.648 | 0.466 | 0.278 | 0.025 | 0.058 | 0.874 | 2.545 | 3.958 | **Medium** |
|  |  | FA7.2 | 31.973 | 8.024 | 23.007 | 5.573 | 0.024 | 0.099 | 0.149 | 3.925 | 0.439 | 0.272 | 0.025 | 0.058 | 0.954 | 2.595 | 3.890 |  |
|  |  | FA7.3 | 30.461 | 7.753 | 23.414 | 5.602 | 0.025 | 0.103 | 0.143 | 3.914 | 0.434 | 0.271 | 0.024 | 0.058 | 0.920 | 2.567 | 3.688 |  |
|  |  | FA7.4 | 30.492 | 7.769 | 22.894 | 5.893 | 0.026 | 0.095 | 0.148 | 3.982 | 0.446 | 0.269 | 0.026 | 0.058 | 0.952 | 2.585 | 3.986 |  |
|  |  | FA7.5 | 30.555 | 7.730 | 22.510 | 5.910 | 0.026 | 0.099 | 0.150 | 3.686 | 0.475 | 0.275 | 0.024 | 0.058 | 0.918 | 2.489 | 3.716 |  |
| **Winter** | **December 6,2017** | WI1.1 | 14.865 | 7.502 | 19.418 | 8.547 | 0.010 | 0.229 | 0.072 | 1.140 | 0.677 | 0.311 | 0.027 | 0.058 | 1.240 | 2.920 | 5.490 |  |
|  |  | WI1.2 | 15.608 | 7.236 | 18.340 | 8.859 | 0.010 | 0.210 | 0.078 | 1.086 | 0.700 | 0.297 | 0.029 | 0.058 | 1.287 | 3.050 | 5.857 |  |
|  |  | WI1.3 | 14.181 | 7.502 | 19.028 | 8.366 | 0.010 | 0.212 | 0.074 | 1.091 | 0.697 | 0.297 | 0.028 | 0.063 | 1.195 | 3.096 | 5.664 |  |
|  |  | WI1.4 | 15.133 | 7.078 | 18.209 | 8.868 | 0.010 | 0.221 | 0.077 | 1.118 | 0.684 | 0.308 | 0.027 | 0.061 | 1.270 | 2.905 | 5.372 |  |
|  |  | WI1.5 | 15.474 | 7.517 | 19.344 | 8.781 | 0.010 | 0.218 | 0.077 | 1.112 | 0.681 | 0.304 | 0.027 | 0.061 | 1.219 | 2.984 | 5.478 |  |
|  |  | WI2.1 | 14.756 | 7.169 | 18.010 | 8.702 | 0.007 | 0.140 | 0.071 | 1.252 | 0.452 | 0.219 | 0.024 | 0.057 | 1.161 | 2.213 | 2.574 | **Low** |
|  |  | WI2.2 | 14.653 | 7.337 | 19.232 | 8.931 | 0.007 | 0.144 | 0.071 | 1.349 | 0.452 | 0.222 | 0.024 | 0.059 | 1.158 | 2.240 | 2.607 |  |
|  |  | WI2.3 | 14.490 | 7.118 | 19.514 | 8.904 | 0.007 | 0.154 | 0.065 | 1.242 | 0.472 | 0.226 | 0.024 | 0.062 | 1.103 | 2.365 | 2.852 |  |
|  |  | WI2.4 | 15.067 | 7.066 | 19.195 | 8.535 | 0.007 | 0.141 | 0.070 | 1.268 | 0.479 | 0.218 | 0.025 | 0.060 | 1.115 | 2.301 | 2.773 |  |
|  |  | WI2.5 | 14.268 | 7.504 | 19.665 | 8.913 | 0.007 | 0.147 | 0.066 | 1.347 | 0.445 | 0.220 | 0.024 | 0.062 | 1.120 | 2.308 | 2.711 |  |
|  |  | WI3.1 | 15.217 | 7.597 | 19.450 | 9.076 | 0.007 | 0.167 | 0.072 | 1.466 | 0.561 | 0.246 | 0.020 | 0.056 | 1.269 | 2.761 | 2.946 |  |
|  |  | WI3.2 | 14.726 | 7.803 | 18.797 | 8.859 | 0.007 | 0.175 | 0.074 | 1.446 | 0.570 | 0.256 | 0.020 | 0.056 | 1.264 | 2.603 | 2.985 |  |
|  |  | WI3.3 | 14.429 | 7.803 | 18.701 | 8.913 | 0.007 | 0.161 | 0.072 | 1.488 | 0.572 | 0.239 | 0.020 | 0.057 | 1.242 | 2.744 | 2.895 |  |
|  |  | WI3.4 | 15.127 | 7.765 | 18.912 | 9.148 | 0.007 | 0.167 | 0.072 | 1.532 | 0.578 | 0.246 | 0.020 | 0.055 | 1.169 | 2.601 | 2.908 |  |
|  |  | WI3.5 | 15.410 | 7.871 | 18.586 | 8.706 | 0.007 | 0.167 | 0.072 | 1.427 | 0.562 | 0.245 | 0.021 | 0.055 | 1.204 | 2.670 | 2.991 |  |
|  |  | WI4.1 | 15.158 | 7.182 | 21.449 | 8.913 | 0.005 | 0.120 | 0.051 | 0.780 | 0.577 | 0.176 | 0.016 | 0.050 | 1.085 | 1.871 | 1.202 |  |
|  |  | WI4.2 | 15.068 | 7.122 | 21.924 | 8.544 | 0.005 | 0.119 | 0.047 | 0.780 | 0.568 | 0.171 | 0.017 | 0.053 | 1.154 | 1.787 | 1.158 |  |
|  |  | WI4.3 | 14.679 | 7.778 | 20.585 | 8.772 | 0.005 | 0.111 | 0.048 | 0.834 | 0.568 | 0.164 | 0.016 | 0.051 | 1.073 | 1.806 | 1.076 |  |
|  |  | WI4.4 | 15.232 | 7.793 | 20.822 | 8.825 | 0.005 | 0.120 | 0.051 | 0.823 | 0.598 | 0.176 | 0.017 | 0.052 | 1.109 | 1.783 | 1.160 |  |
|  |  | WI4.5 | 15.681 | 7.502 | 21.838 | 9.036 | 0.005 | 0.118 | 0.051 | 0.807 | 0.589 | 0.174 | 0.018 | 0.054 | 1.065 | 1.856 | 1.280 |  |
|  |  | WI5.1 | 14.474 | 7.740 | 22.434 | 9.627 | 0.005 | 0.131 | 0.067 | 1.273 | 0.592 | 0.202 | 0.014 | 0.059 | 0.965 | 1.867 | 1.174 |  |
|  |  | WI5.2 | 14.831 | 7.433 | 21.475 | 9.184 | 0.005 | 0.135 | 0.065 | 1.266 | 0.564 | 0.205 | 0.014 | 0.060 | 0.887 | 1.878 | 1.221 |  |
|  |  | WI5.3 | 14.207 | 7.253 | 21.631 | 9.295 | 0.005 | 0.127 | 0.065 | 1.365 | 0.551 | 0.197 | 0.014 | 0.062 | 0.933 | 1.777 | 1.089 |  |
|  |  | WI5.4 | 15.441 | 7.478 | 22.456 | 9.110 | 0.005 | 0.124 | 0.063 | 1.248 | 0.565 | 0.192 | 0.014 | 0.064 | 0.911 | 1.872 | 1.097 |  |
|  |  | WI5.5 | 15.382 | 7.733 | 21.631 | 9.424 | 0.005 | 0.123 | 0.066 | 1.264 | 0.552 | 0.194 | 0.014 | 0.063 | 0.939 | 1.818 | 1.082 |  |
|  |  | WI6.1 | 14.996 | 7.125 | 21.280 | 9.219 | 0.005 | 0.109 | 0.059 | 1.732 | 0.536 | 0.173 | 0.015 | 0.078 | 1.003 | 2.025 | 1.157 |  |
|  |  | WI6.2 | 14.431 | 7.763 | 22.176 | 8.773 | 0.005 | 0.101 | 0.057 | 1.765 | 0.492 | 0.163 | 0.014 | 0.081 | 0.977 | 2.094 | 1.088 |  |
|  |  | WI6.3 | 14.208 | 7.200 | 21.750 | 9.092 | 0.005 | 0.103 | 0.058 | 1.729 | 0.532 | 0.166 | 0.014 | 0.075 | 0.973 | 1.951 | 1.037 |  |
|  |  | WI6.4 | 15.575 | 7.740 | 21.280 | 8.864 | 0.005 | 0.109 | 0.060 | 1.686 | 0.525 | 0.174 | 0.015 | 0.076 | 0.947 | 2.065 | 1.159 |  |
|  |  | WI6.5 | 14.163 | 7.605 | 23.386 | 9.019 | 0.005 | 0.106 | 0.058 | 1.629 | 0.515 | 0.169 | 0.014 | 0.075 | 0.936 | 2.108 | 1.131 |  |
|  |  | WI7.1 | 15.474 | 7.762 | 23.594 | 8.617 | 0.005 | 0.164 | 0.061 | 2.062 | 0.605 | 0.230 | 0.012 | 0.083 | 0.896 | 1.763 | 1.041 |  |
|  |  | WI7.2 | 15.294 | 7.415 | 24.511 | 8.538 | 0.005 | 0.158 | 0.060 | 2.024 | 0.606 | 0.223 | 0.012 | 0.079 | 0.867 | 1.791 | 1.031 |  |
|  |  | WI7.3 | 14.336 | 7.523 | 24.417 | 8.952 | 0.005 | 0.166 | 0.062 | 2.058 | 0.596 | 0.233 | 0.012 | 0.081 | 0.835 | 1.708 | 1.025 |  |
|  |  | WI7.4 | 14.531 | 7.354 | 24.417 | 9.093 | 0.005 | 0.162 | 0.059 | 2.008 | 0.596 | 0.226 | 0.011 | 0.083 | 0.857 | 1.807 | 1.041 |  |
|  |  | WI7.5 | 14.576 | 7.546 | 23.030 | 8.846 | 0.005 | 0.154 | 0.061 | 2.142 | 0.599 | 0.220 | 0.013 | 0.085 | 0.836 | 1.800 | 1.107 |  |
| **Spring** | **March 11,2018** | SP1.1 | 24.237 | 8.016 | 19.713 | 9.386 | 0.022 | 0.447 | 0.168 | 1.606 | 0.884 | 0.637 | 0.039 | 0.086 | 1.132 | 2.970 | 16.276 | **High** |
|  |  | SP1.2 | 24.525 | 7.605 | 18.257 | 9.100 | 0.021 | 0.425 | 0.169 | 1.651 | 0.839 | 0.615 | 0.037 | 0.083 | 1.201 | 2.961 | 14.950 |  |
|  |  | SP1.3 | 24.333 | 7.426 | 18.957 | 8.953 | 0.022 | 0.456 | 0.172 | 1.582 | 0.836 | 0.650 | 0.035 | 0.078 | 1.139 | 3.137 | 15.985 |  |
|  |  | SP1.4 | 23.040 | 7.597 | 19.505 | 8.851 | 0.021 | 0.465 | 0.177 | 1.664 | 0.912 | 0.663 | 0.035 | 0.079 | 1.105 | 3.199 | 16.726 |  |
|  |  | SP1.5 | 22.824 | 8.001 | 19.524 | 9.533 | 0.021 | 0.434 | 0.183 | 1.638 | 0.918 | 0.638 | 0.038 | 0.085 | 1.164 | 3.128 | 16.966 |  |
|  |  | SP2.1 | 24.603 | 7.784 | 18.579 | 8.530 | 0.021 | 0.430 | 0.173 | 1.648 | 0.820 | 0.624 | 0.047 | 0.080 | 1.043 | 2.714 | 17.792 |  |
|  |  | SP2.2 | 24.819 | 7.452 | 18.239 | 8.790 | 0.022 | 0.425 | 0.170 | 1.608 | 0.773 | 0.617 | 0.047 | 0.074 | 1.015 | 2.736 | 17.464 |  |
|  |  | SP2.3 | 23.932 | 8.001 | 19.788 | 9.032 | 0.023 | 0.451 | 0.182 | 1.581 | 0.752 | 0.655 | 0.048 | 0.076 | 1.023 | 2.841 | 19.797 |  |
|  |  | SP2.4 | 23.237 | 7.745 | 19.165 | 8.736 | 0.022 | 0.444 | 0.181 | 1.563 | 0.772 | 0.647 | 0.046 | 0.082 | 1.028 | 2.841 | 18.989 |  |
|  |  | SP2.5 | 23.572 | 7.877 | 18.068 | 9.247 | 0.021 | 0.437 | 0.185 | 1.582 | 0.801 | 0.643 | 0.046 | 0.082 | 1.003 | 2.850 | 18.647 |  |
|  |  | SP3.1 | 23.952 | 7.549 | 20.156 | 9.639 | 0.019 | 0.381 | 0.162 | 1.325 | 0.761 | 0.562 | 0.068 | 0.079 | 1.097 | 3.048 | 26.028 |  |
|  |  | SP3.2 | 24.549 | 7.845 | 18.949 | 8.850 | 0.018 | 0.378 | 0.163 | 1.247 | 0.741 | 0.559 | 0.068 | 0.082 | 1.057 | 3.048 | 25.892 |  |
|  |  | SP3.3 | 23.187 | 7.852 | 20.117 | 8.850 | 0.020 | 0.362 | 0.172 | 1.299 | 0.774 | 0.553 | 0.070 | 0.083 | 1.082 | 2.883 | 24.652 |  |
|  |  | SP3.4 | 23.474 | 7.977 | 20.453 | 9.391 | 0.019 | 0.382 | 0.176 | 1.242 | 0.764 | 0.577 | 0.065 | 0.082 | 1.082 | 2.868 | 23.949 |  |
|  |  | SP3.5 | 24.931 | 7.494 | 19.998 | 9.373 | 0.019 | 0.371 | 0.169 | 1.314 | 0.733 | 0.560 | 0.068 | 0.081 | 1.115 | 2.865 | 24.077 |  |
|  |  | SP4.1 | 23.974 | 7.876 | 21.967 | 9.419 | 0.012 | 0.344 | 0.123 | 0.934 | 0.677 | 0.480 | 0.028 | 0.082 | 0.865 | 2.132 | 6.413 | **Medium** |
|  |  | SP4.2 | 23.663 | 7.587 | 22.118 | 8.674 | 0.013 | 0.343 | 0.122 | 0.894 | 0.696 | 0.478 | 0.030 | 0.084 | 0.847 | 2.162 | 6.922 |  |
|  |  | SP4.3 | 22.968 | 8.125 | 21.492 | 8.925 | 0.013 | 0.352 | 0.120 | 0.926 | 0.729 | 0.485 | 0.030 | 0.088 | 0.841 | 2.218 | 7.056 |  |
|  |  | SP4.4 | 24.573 | 8.063 | 22.010 | 8.907 | 0.013 | 0.355 | 0.112 | 0.886 | 0.698 | 0.480 | 0.028 | 0.088 | 0.833 | 2.255 | 6.829 |  |
|  |  | SP4.5 | 25.148 | 7.821 | 22.075 | 8.584 | 0.013 | 0.339 | 0.120 | 0.883 | 0.718 | 0.472 | 0.028 | 0.087 | 0.834 | 2.143 | 6.296 |  |
|  |  | SP5.1 | 24.559 | 7.372 | 23.575 | 8.910 | 0.012 | 0.333 | 0.117 | 1.637 | 0.714 | 0.462 | 0.039 | 0.090 | 0.844 | 2.222 | 8.815 |  |
|  |  | SP5.2 | 24.990 | 7.970 | 22.526 | 8.586 | 0.012 | 0.351 | 0.118 | 1.605 | 0.713 | 0.482 | 0.039 | 0.087 | 0.808 | 2.188 | 9.124 |  |
|  |  | SP5.3 | 23.337 | 7.442 | 22.230 | 8.437 | 0.012 | 0.326 | 0.114 | 1.557 | 0.695 | 0.452 | 0.038 | 0.089 | 0.813 | 2.199 | 8.388 |  |
|  |  | SP5.4 | 24.559 | 7.783 | 22.960 | 8.875 | 0.013 | 0.331 | 0.118 | 1.659 | 0.697 | 0.462 | 0.040 | 0.089 | 0.828 | 2.177 | 8.988 |  |
|  |  | SP5.5 | 24.655 | 7.698 | 22.504 | 8.419 | 0.012 | 0.352 | 0.118 | 1.557 | 0.712 | 0.482 | 0.039 | 0.092 | 0.813 | 2.115 | 8.798 |  |
|  |  | SP6.1 | 23.423 | 7.764 | 22.259 | 8.923 | 0.011 | 0.361 | 0.120 | 1.919 | 0.756 | 0.492 | 0.044 | 0.084 | 0.911 | 2.584 | 12.300 | **High** |
|  |  | SP6.2 | 22.920 | 7.741 | 22.396 | 8.951 | 0.011 | 0.357 | 0.113 | 1.995 | 0.750 | 0.481 | 0.043 | 0.082 | 0.982 | 2.389 | 10.951 |  |
|  |  | SP6.3 | 24.285 | 7.819 | 21.801 | 9.539 | 0.011 | 0.361 | 0.115 | 1.957 | 0.721 | 0.487 | 0.044 | 0.085 | 0.964 | 2.522 | 11.993 |  |
|  |  | SP6.4 | 24.836 | 7.998 | 23.518 | 9.264 | 0.011 | 0.356 | 0.122 | 1.872 | 0.776 | 0.490 | 0.046 | 0.080 | 0.907 | 2.411 | 11.974 |  |
|  |  | SP6.5 | 25.028 | 7.764 | 21.915 | 8.933 | 0.011 | 0.334 | 0.121 | 1.986 | 0.719 | 0.466 | 0.045 | 0.082 | 0.900 | 2.529 | 11.784 |  |
|  |  | SP7.1 | 23.764 | 7.599 | 22.965 | 8.869 | 0.010 | 0.341 | 0.123 | 2.284 | 0.805 | 0.473 | 0.043 | 0.088 | 0.696 | 1.895 | 8.632 | **Medium** |
|  |  | SP7.2 | 24.771 | 7.686 | 23.937 | 9.102 | 0.010 | 0.327 | 0.125 | 2.210 | 0.785 | 0.462 | 0.042 | 0.088 | 0.735 | 1.874 | 8.152 |  |
|  |  | SP7.3 | 24.268 | 7.948 | 24.482 | 8.896 | 0.010 | 0.335 | 0.132 | 2.371 | 0.767 | 0.477 | 0.041 | 0.085 | 0.683 | 1.925 | 8.325 |  |
|  |  | SP7.4 | 24.580 | 8.234 | 22.894 | 8.932 | 0.010 | 0.332 | 0.124 | 2.309 | 0.784 | 0.466 | 0.044 | 0.085 | 0.701 | 1.839 | 8.434 |  |
|  |  | SP7.5 | 24.651 | 7.924 | 23.676 | 9.308 | 0.010 | 0.349 | 0.128 | 2.254 | 0.774 | 0.487 | 0.042 | 0.087 | 0.728 | 1.919 | 8.752 |  |

| **Sampling Sites** | | |
| --- | --- | --- |
| **Site** | **N** | **E** |
| M1 | 21.835654 | 108.54385 |
| M2 | 21.822243 | 108.54238 |
| M3 | 21.801924 | 108.54155 |
| M4 | 21.742779 | 108.55918 |
| M5 | 21.736457 | 108.57424 |
| M6 | 21.71962 | 108.5756 |
| M7 | 21.710154 | 108.5841 |

**Chl *a*:** Chlorophyll A;

**DIN:** Dissolved Inorganic Nitrogen;

**TDN:** Total Dissolved Nitrogen;

**DIP:** Dissolved Inorganic Phosphorus;

**TDP:** Total Dissolved Phosphorus;

**TOC:** Total Organic Carbon;

**COD:** Chemical Oxygen Demand;

**EI:** Eutrophication Index;

**Table S2 Number of sequences and OTUs and alpha diversity estimates**

| **Seasons** | **Sites** | **OTUs** | **No. of Seq** | **Shannon** | **Simpson** | **Chao1** | **Coverage (%)** |
| --- | --- | --- | --- | --- | --- | --- | --- |
| **Spring** | SP1.1 | 69 | 15893 | 0.326 | 0.090 | 112.5 | 99.81 |
|  | SP1.2 | 69 | 15884 | 0.330 | 0.091 | 105.3 | 99.81 |
|  | SP1.3 | 71 | 15896 | 0.330 | 0.091 | 127.1 | 99.79 |
|  | SP1.4 | 67 | 15886 | 0.334 | 0.092 | 104.8 | 99.82 |
|  | SP1.5 | 70 | 15878 | 0.328 | 0.091 | 169.2 | 99.78 |
|  | SP2.1 | 109 | 23481 | 0.482 | 0.141 | 347.5 | 99.77 |
|  | SP2.2 | 108 | 23481 | 0.477 | 0.139 | 304.9 | 99.77 |
|  | SP2.3 | 105 | 23481 | 0.478 | 0.140 | 246.0 | 99.80 |
|  | SP2.4 | 105 | 23480 | 0.477 | 0.139 | 188.2 | 99.80 |
|  | SP2.5 | 108 | 23481 | 0.485 | 0.141 | 255.0 | 99.79 |
|  | SP3.1 | 51 | 23250 | 0.073 | 0.016 | 132.2 | 99.88 |
|  | SP3.2 | 54 | 23254 | 0.077 | 0.017 | 104.8 | 99.88 |
|  | SP3.3 | 53 | 23253 | 0.073 | 0.016 | 125.5 | 99.87 |
|  | SP3.4 | 54 | 23254 | 0.069 | 0.015 | 126.5 | 99.87 |
|  | SP3.5 | 54 | 23254 | 0.073 | 0.016 | 131.5 | 99.87 |
|  | SP4.1 | 50 | 19446 | 0.115 | 0.029 | 73.0 | 99.88 |
|  | SP4.2 | 52 | 19439 | 0.116 | 0.029 | 94.0 | 99.86 |
|  | SP4.3 | 50 | 19435 | 0.113 | 0.028 | 89.0 | 99.86 |
|  | SP4.4 | 52 | 19432 | 0.116 | 0.028 | 81.5 | 99.87 |
|  | SP4.5 | 52 | 19440 | 0.118 | 0.029 | 81.5 | 99.87 |
|  | SP5.1 | 94 | 9145 | 1.072 | 0.349 | 298.2 | 99.45 |
|  | SP5.2 | 85 | 9161 | 1.059 | 0.346 | 177.6 | 99.57 |
|  | SP5.3 | 90 | 9148 | 1.073 | 0.349 | 195.1 | 99.52 |
|  | SP5.4 | 91 | 9148 | 1.082 | 0.352 | 209.3 | 99.52 |
|  | SP5.5 | 86 | 9164 | 1.069 | 0.347 | 172.7 | 99.56 |
|  | SP6.1 | 88 | 8638 | 0.836 | 0.245 | 123.8 | 99.64 |
|  | SP6.2 | 86 | 8649 | 0.838 | 0.246 | 144.7 | 99.62 |
|  | SP6.3 | 88 | 8643 | 0.824 | 0.241 | 146.7 | 99.62 |
|  | SP6.4 | 82 | 8635 | 0.823 | 0.241 | 109.1 | 99.70 |
|  | SP6.5 | 85 | 8638 | 0.821 | 0.241 | 136.7 | 99.64 |
|  | SP7.1 | 76 | 5371 | 1.898 | 0.645 | 130.2 | 99.52 |
|  | SP7.2 | 79 | 5348 | 1.900 | 0.646 | 137.0 | 99.46 |
|  | SP7.3 | 77 | 5367 | 1.897 | 0.645 | 127.1 | 99.50 |
|  | SP7.4 | 79 | 5362 | 1.900 | 0.645 | 137.0 | 99.46 |
|  | SP7.5 | 79 | 5374 | 1.899 | 0.646 | 137.0 | 99.46 |
| **Summer** | SU1.1 | 80 | 8874 | 0.344 | 0.086 | 191.0 | 99.58 |
|  | SU1.2 | 78 | 8865 | 0.357 | 0.089 | 166.0 | 99.63 |
|  | SU1.3 | 83 | 8879 | 0.355 | 0.089 | 239.0 | 99.55 |
|  | SU1.4 | 84 | 8870 | 0.363 | 0.090 | 214.0 | 99.55 |
|  | SU1.5 | 86 | 8901 | 0.360 | 0.090 | 181.7 | 99.53 |
|  | SU2.1 | 87 | 8906 | 0.791 | 0.233 | 108.9 | 99.70 |
|  | SU2.2 | 85 | 8913 | 0.781 | 0.230 | 120.8 | 99.65 |
|  | SU2.3 | 82 | 8911 | 0.782 | 0.232 | 118.3 | 99.66 |
|  | SU2.4 | 85 | 8902 | 0.777 | 0.229 | 114.0 | 99.67 |
|  | SU2.5 | 87 | 8915 | 0.776 | 0.229 | 127.6 | 99.63 |
|  | SU3.1 | 119 | 10043 | 1.726 | 0.565 | 188.5 | 99.57 |
|  | SU3.2 | 120 | 10039 | 1.721 | 0.565 | 232.8 | 99.52 |
|  | SU3.3 | 120 | 10030 | 1.717 | 0.563 | 242.5 | 99.50 |
|  | SU3.4 | 124 | 10046 | 1.722 | 0.563 | 251.5 | 99.49 |
|  | SU3.5 | 125 | 10019 | 1.712 | 0.561 | 194.2 | 99.51 |
|  | SU4.1 | 125 | 11248 | 1.250 | 0.365 | 203.8 | 99.61 |
|  | SU4.2 | 125 | 11244 | 1.254 | 0.365 | 192.4 | 99.65 |
|  | SU4.3 | 121 | 11231 | 1.258 | 0.367 | 176.5 | 99.67 |
|  | SU4.4 | 126 | 11245 | 1.252 | 0.365 | 208.1 | 99.62 |
|  | SU4.5 | 126 | 11227 | 1.244 | 0.363 | 216.0 | 99.60 |
|  | SU5.1 | 88 | 11737 | 0.472 | 0.133 | 198.0 | 99.62 |
|  | SU5.2 | 89 | 11728 | 0.473 | 0.133 | 183.6 | 99.62 |
|  | SU5.3 | 89 | 11726 | 0.466 | 0.130 | 179.3 | 99.63 |
|  | SU5.4 | 90 | 11734 | 0.466 | 0.130 | 262.5 | 99.61 |
|  | SU5.5 | 91 | 11739 | 0.468 | 0.131 | 221.7 | 99.58 |
|  | SU6.1 | 91 | 13600 | 1.044 | 0.491 | 169.3 | 99.69 |
|  | SU6.2 | 91 | 13600 | 1.040 | 0.490 | 160.5 | 99.68 |
|  | SU6.3 | 87 | 13599 | 1.036 | 0.487 | 148.8 | 99.71 |
|  | SU6.4 | 85 | 13601 | 1.036 | 0.489 | 148.9 | 99.72 |
|  | SU6.5 | 88 | 13601 | 1.029 | 0.486 | 174.7 | 99.71 |
|  | SU7.1 | 74 | 3947 | 2.042 | 0.690 | 124.1 | 99.32 |
|  | SU7.2 | 75 | 3929 | 2.043 | 0.690 | 129.0 | 99.29 |
|  | SU7.3 | 74 | 3920 | 2.044 | 0.690 | 121.3 | 99.29 |
|  | SU7.4 | 74 | 3942 | 2.041 | 0.689 | 117.9 | 99.32 |
|  | SU7.5 | 75 | 3941 | 2.048 | 0.691 | 122.3 | 99.29 |
| **Fall** | FA1.1 | 69 | 9222 | 0.851 | 0.304 | 109.6 | 99.72 |
|  | FA1.2 | 71 | 9221 | 0.860 | 0.306 | 118.3 | 99.70 |
|  | FA1.3 | 72 | 9223 | 0.853 | 0.306 | 122.8 | 99.69 |
|  | FA1.4 | 68 | 9222 | 0.858 | 0.306 | 114.4 | 99.72 |
|  | FA1.5 | 69 | 9221 | 0.852 | 0.304 | 101.5 | 99.72 |
|  | FA2.1 | 66 | 9275 | 0.747 | 0.231 | 80.3 | 99.80 |
|  | FA2.2 | 66 | 9266 | 0.755 | 0.235 | 96.0 | 99.77 |
|  | FA2.3 | 67 | 9266 | 0.754 | 0.234 | 122.2 | 99.74 |
|  | FA2.4 | 68 | 9272 | 0.769 | 0.238 | 85.1 | 99.80 |
|  | FA2.5 | 70 | 9272 | 0.755 | 0.233 | 85.0 | 99.77 |
|  | FA3.1 | 78 | 8465 | 0.869 | 0.284 | 183.0 | 99.57 |
|  | FA3.2 | 83 | 8466 | 0.889 | 0.288 | 194.4 | 99.53 |
|  | FA3.3 | 86 | 8466 | 0.895 | 0.291 | 160.1 | 99.54 |
|  | FA3.4 | 79 | 8466 | 0.893 | 0.291 | 219.6 | 99.55 |
|  | FA3.5 | 77 | 8467 | 0.886 | 0.288 | 139.0 | 99.62 |
|  | FA4.1 | 67 | 10418 | 0.952 | 0.336 | 91.4 | 99.82 |
|  | FA4.2 | 70 | 10410 | 0.947 | 0.332 | 108.5 | 99.79 |
|  | FA4.3 | 67 | 10424 | 0.943 | 0.332 | 92.5 | 99.83 |
|  | FA4.4 | 71 | 10414 | 0.955 | 0.335 | 113.2 | 99.78 |
|  | FA4.5 | 68 | 10421 | 0.951 | 0.335 | 94.3 | 99.80 |
|  | FA5.1 | 92 | 8911 | 0.973 | 0.341 | 117.6 | 99.66 |
|  | FA5.2 | 97 | 8915 | 0.972 | 0.340 | 142.0 | 99.60 |
|  | FA5.3 | 90 | 8910 | 0.959 | 0.337 | 125.2 | 99.63 |
|  | FA5.4 | 92 | 8908 | 0.966 | 0.340 | 166.0 | 99.58 |
|  | FA5.5 | 95 | 8911 | 0.957 | 0.336 | 162.4 | 99.56 |
|  | FA6.1 | 59 | 8238 | 0.770 | 0.240 | 76.0 | 99.79 |
|  | FA6.2 | 62 | 8237 | 0.763 | 0.239 | 90.9 | 99.73 |
|  | FA6.3 | 64 | 8239 | 0.779 | 0.243 | 85.0 | 99.75 |
|  | FA6.4 | 63 | 8234 | 0.767 | 0.239 | 82.1 | 99.74 |
|  | FA6.5 | 59 | 8235 | 0.761 | 0.238 | 97.0 | 99.76 |
|  | FA7.1 | 93 | 20032 | 0.836 | 0.262 | 156.1 | 99.80 |
|  | FA7.2 | 93 | 20025 | 0.834 | 0.261 | 145.0 | 99.80 |
|  | FA7.3 | 93 | 20011 | 0.831 | 0.261 | 168.3 | 99.79 |
|  | FA7.4 | 95 | 19974 | 0.837 | 0.262 | 164.5 | 99.78 |
|  | FA7.5 | 97 | 19987 | 0.846 | 0.264 | 166.5 | 99.78 |
| **Winter** | WI1.1 | 68 | 5191 | 1.445 | 0.483 | 105.5 | 99.52 |
|  | WI1.2 | 67 | 5204 | 1.446 | 0.484 | 97.7 | 99.54 |
|  | WI1.3 | 66 | 5198 | 1.429 | 0.477 | 94.1 | 99.56 |
|  | WI1.4 | 66 | 5229 | 1.437 | 0.481 | 96.7 | 99.54 |
|  | WI1.5 | 67 | 5198 | 1.442 | 0.481 | 95.1 | 99.56 |
|  | WI2.1 | 91 | 15985 | 0.889 | 0.307 | 120.8 | 99.78 |
|  | WI2.2 | 90 | 15971 | 0.892 | 0.307 | 129.7 | 99.78 |
|  | WI2.3 | 87 | 15975 | 0.889 | 0.308 | 114.4 | 99.81 |
|  | WI2.4 | 90 | 15974 | 0.894 | 0.310 | 187.5 | 99.75 |
|  | WI2.5 | 91 | 15972 | 0.898 | 0.310 | 124.0 | 99.79 |
|  | WI3.1 | 106 | 21604 | 0.706 | 0.228 | 185.7 | 99.76 |
|  | WI3.2 | 109 | 21591 | 0.718 | 0.232 | 182.7 | 99.76 |
|  | WI3.3 | 107 | 21590 | 0.711 | 0.228 | 172.3 | 99.77 |
|  | WI3.4 | 108 | 21595 | 0.707 | 0.229 | 203.4 | 99.75 |
|  | WI3.5 | 105 | 21599 | 0.711 | 0.229 | 168.6 | 99.78 |
|  | WI4.1 | 97 | 16422 | 0.852 | 0.291 | 154.4 | 99.74 |
|  | WI4.2 | 102 | 16419 | 0.851 | 0.290 | 185.2 | 99.71 |
|  | WI4.3 | 99 | 16414 | 0.845 | 0.289 | 202.5 | 99.72 |
|  | WI4.4 | 96 | 16414 | 0.848 | 0.290 | 167.8 | 99.74 |
|  | WI4.5 | 99 | 16418 | 0.858 | 0.292 | 193.1 | 99.72 |
|  | WI5.1 | 110 | 24182 | 0.948 | 0.315 | 167.0 | 99.84 |
|  | WI5.2 | 109 | 24185 | 0.947 | 0.316 | 250.4 | 99.81 |
|  | WI5.3 | 111 | 24180 | 0.951 | 0.317 | 169.6 | 99.83 |
|  | WI5.4 | 111 | 24188 | 0.956 | 0.318 | 205.6 | 99.82 |
|  | WI5.5 | 111 | 24183 | 0.952 | 0.318 | 162.3 | 99.83 |
|  | WI6.1 | 79 | 11709 | 1.555 | 0.655 | 112.2 | 99.74 |
|  | WI6.2 | 77 | 11709 | 1.552 | 0.655 | 118.3 | 99.73 |
|  | WI6.3 | 80 | 11711 | 1.561 | 0.656 | 146.1 | 99.70 |
|  | WI6.4 | 77 | 11709 | 1.556 | 0.656 | 115.8 | 99.74 |
|  | WI6.5 | 75 | 11709 | 1.548 | 0.653 | 108.8 | 99.75 |
|  | WI7.1 | 86 | 9335 | 1.198 | 0.395 | 113.0 | 99.70 |
|  | WI7.2 | 88 | 9331 | 1.199 | 0.393 | 128.6 | 99.69 |
|  | WI7.3 | 87 | 9339 | 1.197 | 0.394 | 132.1 | 99.69 |
|  | WI7.4 | 92 | 9332 | 1.200 | 0.394 | 158.1 | 99.62 |
|  | WI7.5 | 92 | 9334 | 1.199 | 0.393 | 150.7 | 99.65 |

**Table S3 Spearman correlations between the Alpha-diversity (OTU level) and environmental factors**

|  | **Shannon** | **Simpson** | **Observed OTUs Num.** | **Chao1** |
| --- | --- | --- | --- | --- |
| **Temp** | -0.07 | -0.08 | -0.18 * | -0.1 |
| **pH** | -0.09 | -0.09 | 0.07 | 0.12 |
| **Salinity** | 0.28 *** | 0.29 *** | 0.18 * | 0.15 |
| **DO** | -0.09 | -0.1 | 0.04 | 0.06 |
| **NO_2_^-^** | -0.4 *** | -0.41 *** | -0.36 *** | -0.25 ** |
| **NO_3_^-^** | -0.28 *** | -0.31 *** | -0.06 | 0.17 * |
| **NH_4_^+^** | -0.28 *** | -0.26 ** | -0.16 | -0.03 |
| **Chl *a*** | 0.03 | 0.02 | 0 | 0.02 |
| **TDN** | -0.38 *** | -0.39 *** | -0.5 *** | -0.24 ** |
| **DIN** | -0.31 *** | -0.33 *** | -0.1 | 0.12 |
| **DIP** | -0.25 ** | -0.24 ** | -0.45 *** | -0.35 *** |
| **TDP** | -0.14 | -0.13 | -0.28 *** | -0.19 * |
| **TOC** | -0.12 | -0.12 | -0.09 | -0.07 |
| **COD** | -0.25 ** | -0.26 ** | -0.17 * | -0.02 |

*, *p* < 0.05; **, *p* < 0.01; ***, *p* < 0.001

**Table S4 The dbRDA results**

|  | **CAP1** | **CAP2** | **r^2^** | **Pr(>r)** |
| --- | --- | --- | --- | --- |
| **Temp** | 0.967 | -0.253 | 0.079 | 0.001 *** |
| **pH** | -0.370 | -0.929 | 0.181 | 0.001 *** |
| **Salinity** | -0.570 | -0.822 | 0.156 | 0.001 *** |
| **DO** | -0.963 | 0.270 | 0.161 | 0.001 *** |
| **NO_2_^-^** | 0.567 | 0.824 | 0.158 | 0.001 *** |
| **NO_3_^-^** | 0.723 | -0.691 | 0.067 | 0.007 ** |
| **NH_4_^+^** | -0.565 | -0.825 | 0.105 | 0.002 *** |
| **Chl *a*** | 0.801 | 0.598 | 0.138 | 0.001 *** |
| **TDN** | 0.482 | 0.876 | 0.122 | 0.001*** |
| **DIN** | 0.614 | -0.790 | 0.052 | 0.028 * |
| **DIP** | -0.979 | 0.204 | 0.011 | 0.475 |
| **TDP** | -0.417 | 0.909 | 0.145 | 0.001 *** |
| **TOC** | 0.707 | 0.707 | 0.129 | 0.001 *** |
| **COD** | 0.896 | 0.445 | 0.046 | 0.029* |

*, *p* < 0.05; **, *p* < 0.01; ***, *p* < 0.001


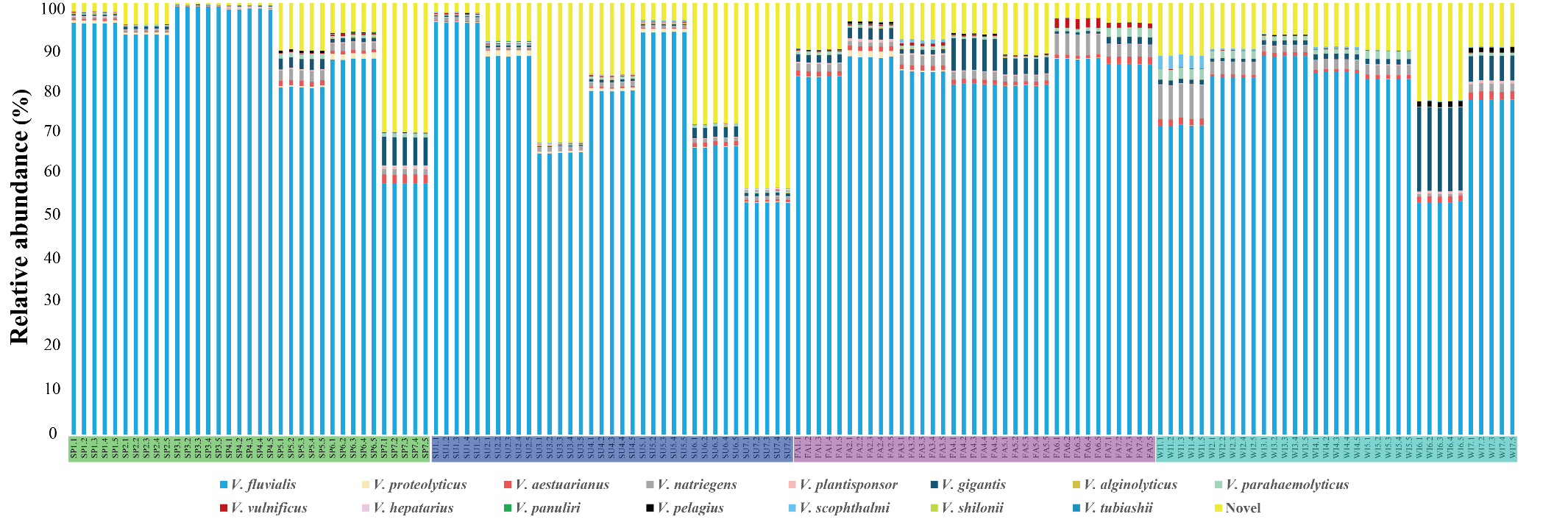


**Figure S1: The** **relative abundance (%) of *Vibrio* community compositions (>1%) at each sample in four seasons.**


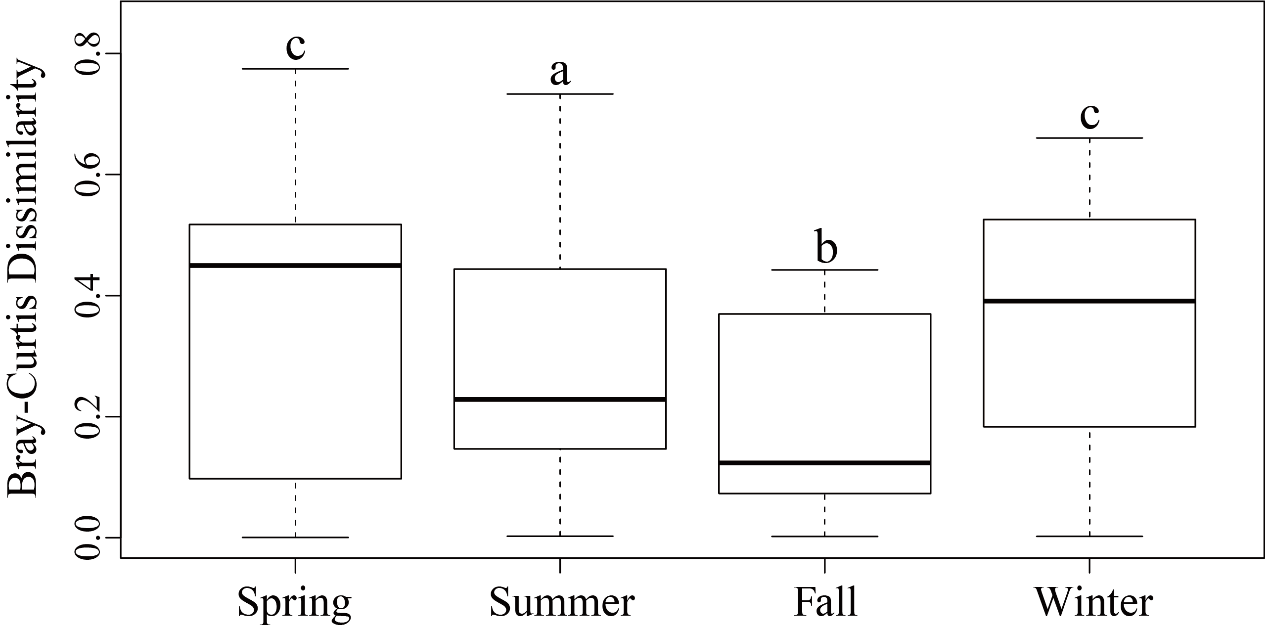


**Figure S2: The Bray-Curtis dissimilarity of the *Vibrio* community in four seasons is presented using a boxplot. Lowercase letters shared in common among the seasons denote no significant difference (*p* > 0.05) determined by an ANOVA with post hoc Tukey's HSD test.**


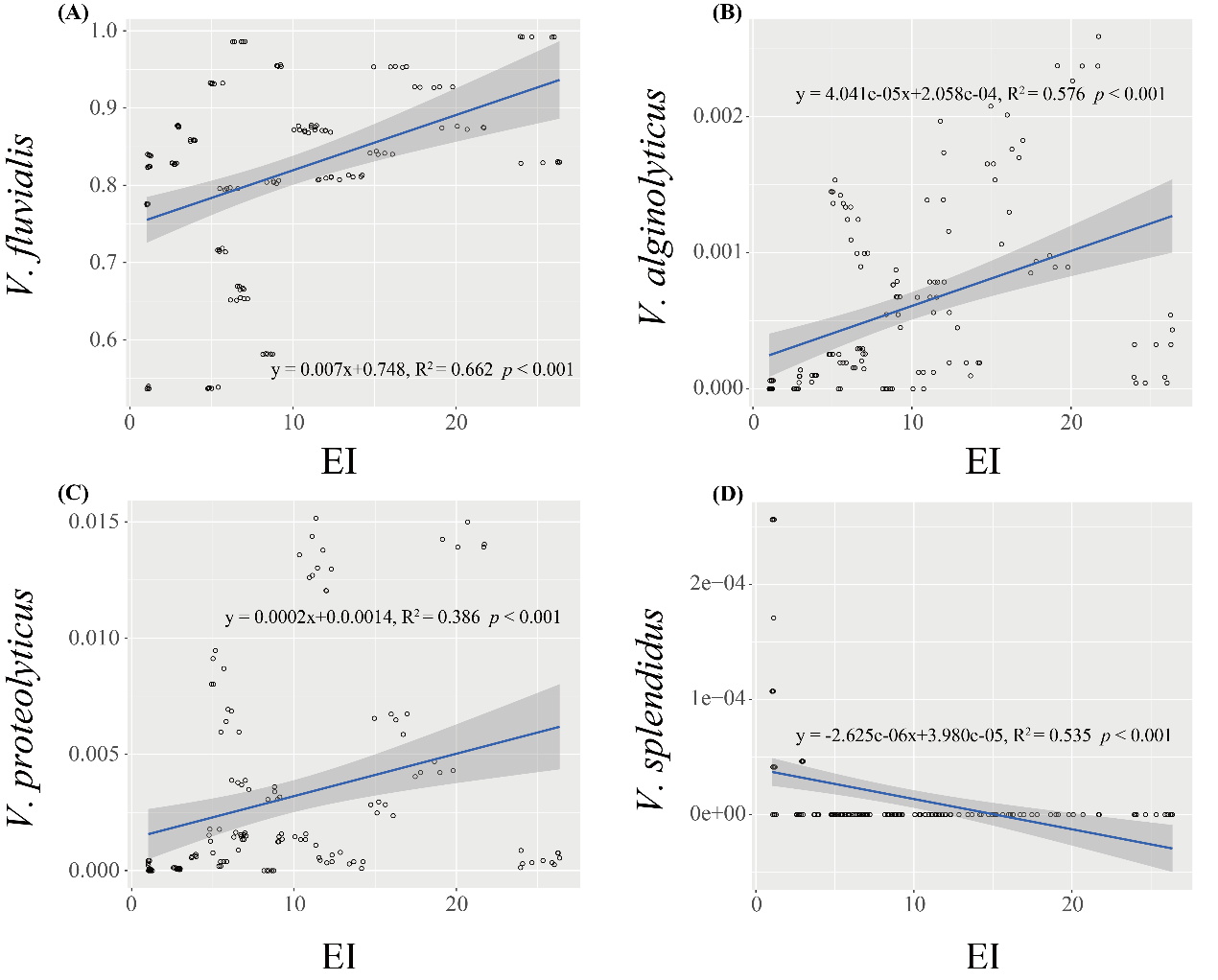


**Figure S3: The regression between EI variable and the main *Vibrio* species (*V. fluvialis*, *V. alginolyticus*, *V. proteolyticus*, and *V. splendidus*) according to random forest results.**


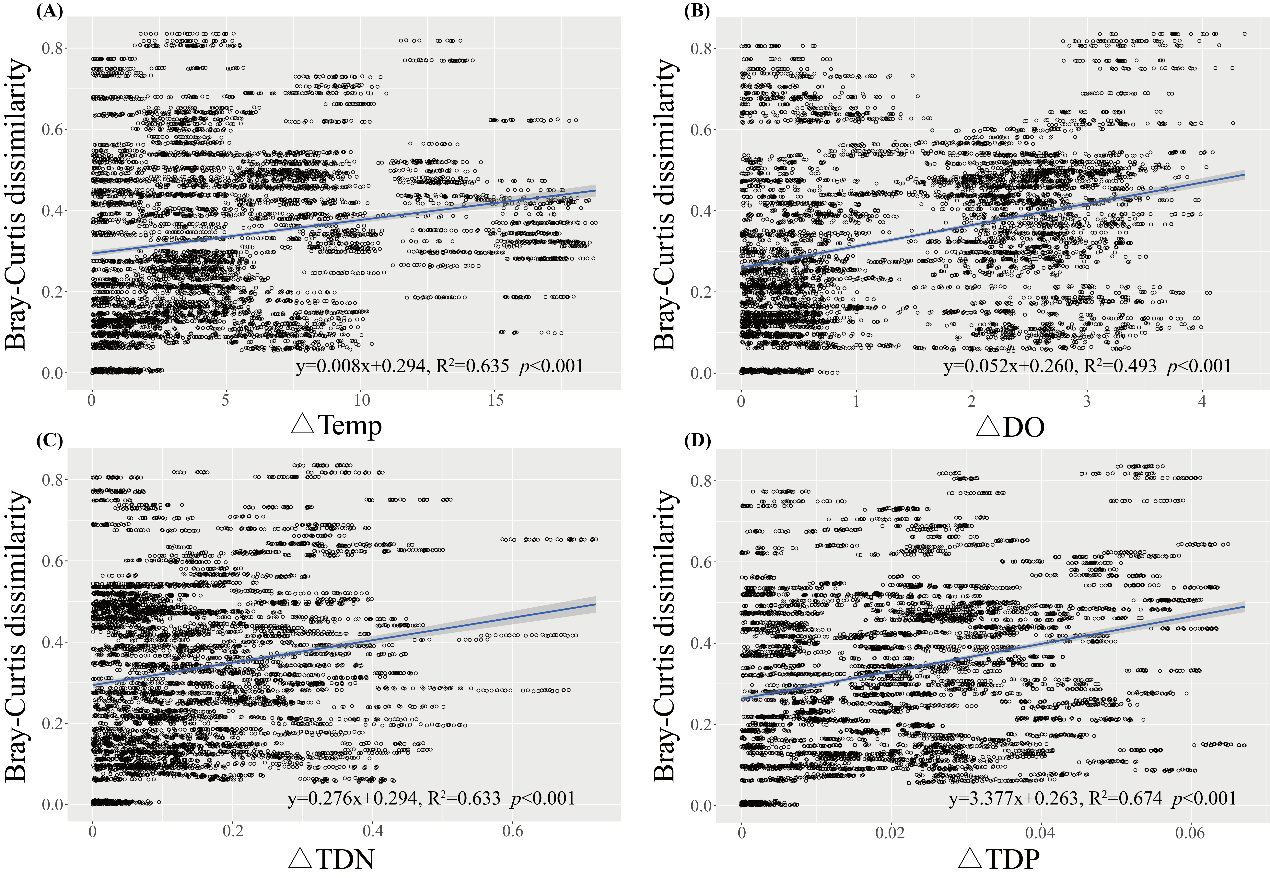


**Figure S4: The regression between Bray-Curtis dissimilarity and main environmental variables. The black line represents the linear regression and *P* values were calculated to indicate significant differences.**
